# Supplementary figures and images for: Identity-by-descent analyses for measuring population dynamics and selection in recombining pathogens
Source: PLoS Genet. 2018 May 23;14(5):e1007279. doi: 10.1371/journal.pgen.1007279 (PMC5988311; doi:10.1371/journal.pgen.1007279)

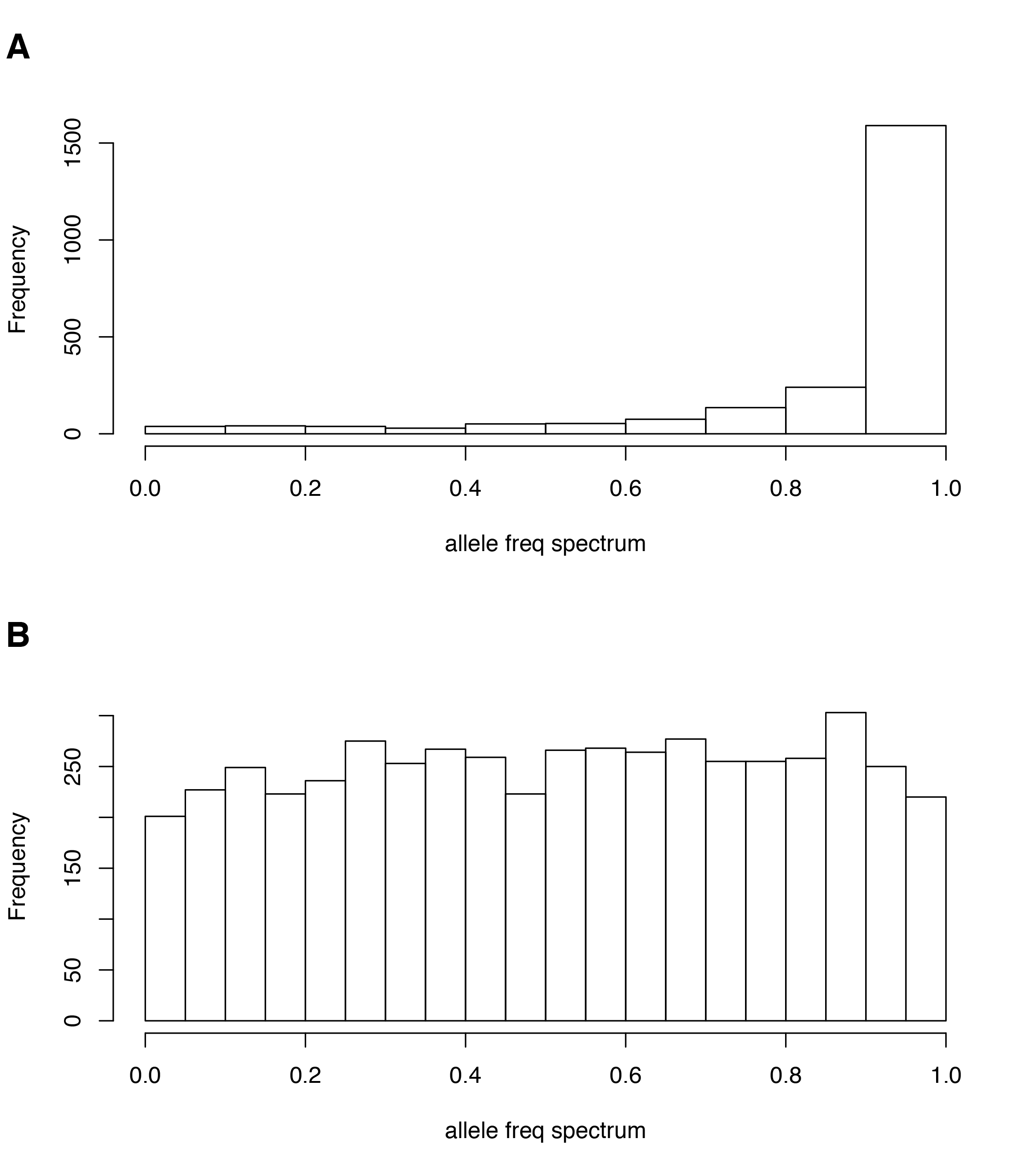

Supplement: S1 Fig — (A) The allele frequency spectrum for the simulated P. falciparum data, which was based on allele frequencies calculated from Pf3k Cambodian isolates. (B) The allele frequency spectrum for the simulated sequencing data based on a uniform allele frequency distribution. SNPs will MAF < 1% have been removed. (TIF) [file pgen.1007279.s001.tif]

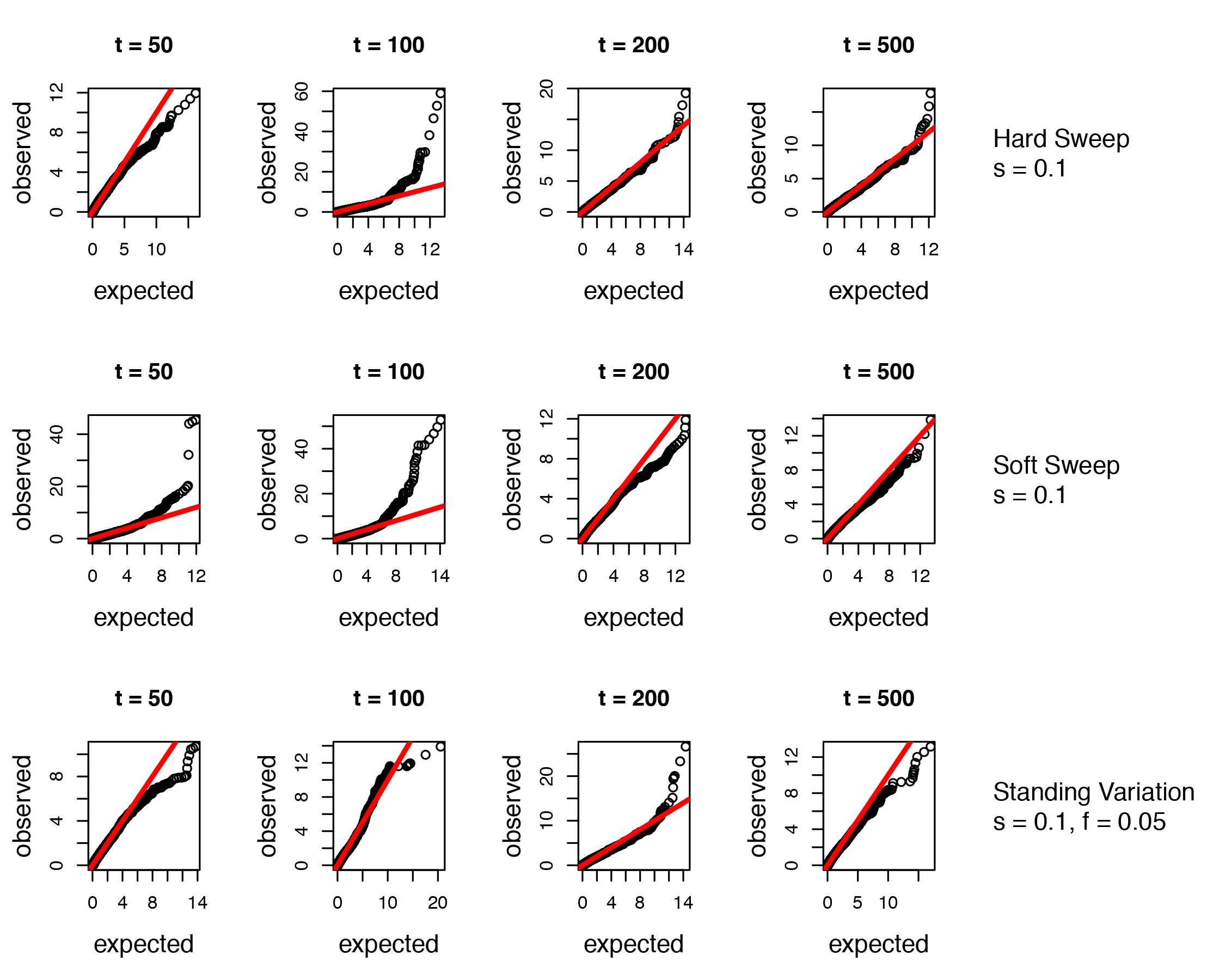

Supplement: S2 Fig — These plots correspond to one replicate of each scenario, where t is the number of generations since the sweep was introduced. (TIF) [file pgen.1007279.s002.tif]

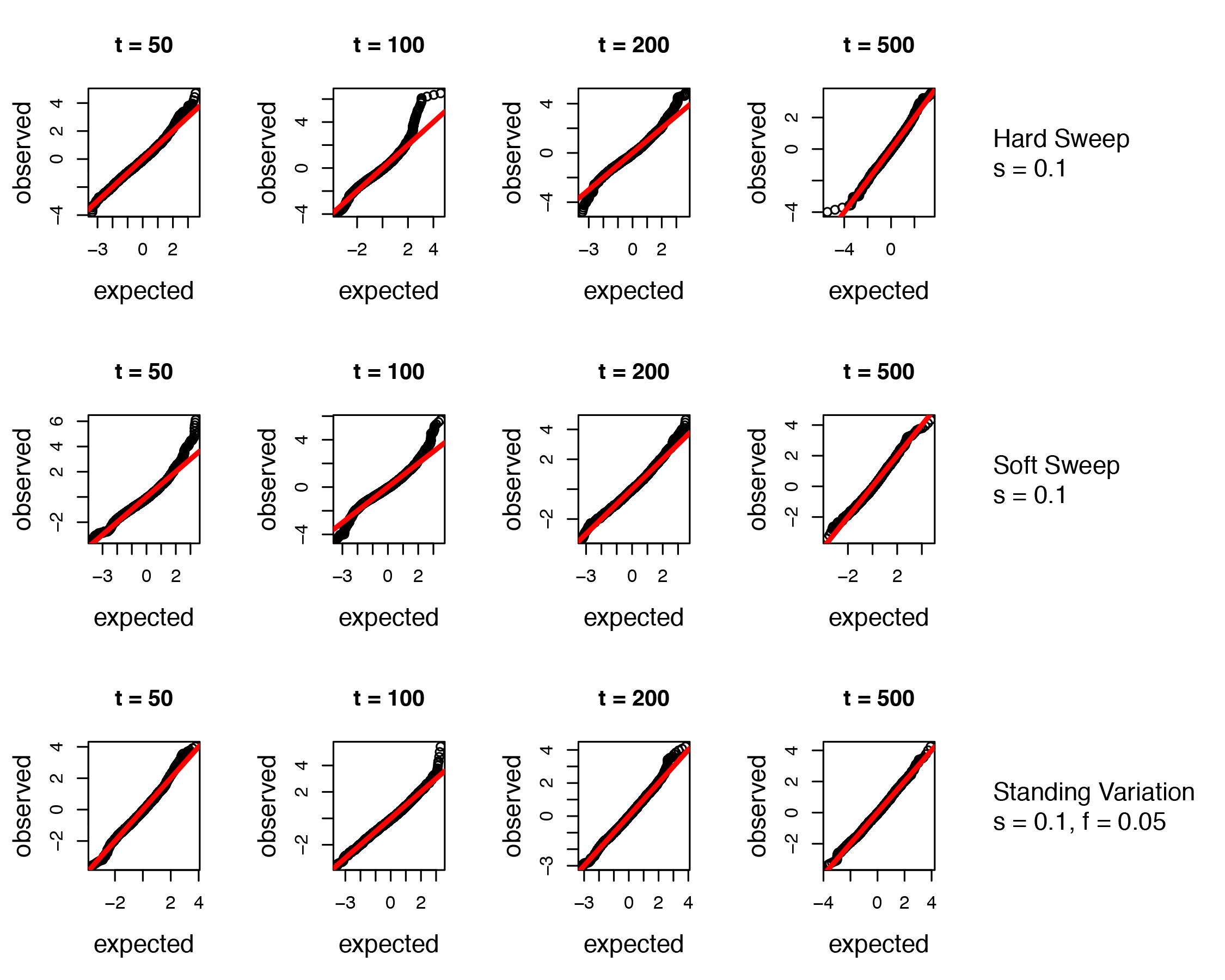

Supplement: S3 Fig — These plots correspond to one replicate of each scenario, where t is the number of generations since the sweep was introduced. The same replicates were used in these figures as in S2 Fig. (TIF) [file pgen.1007279.s003.tif]

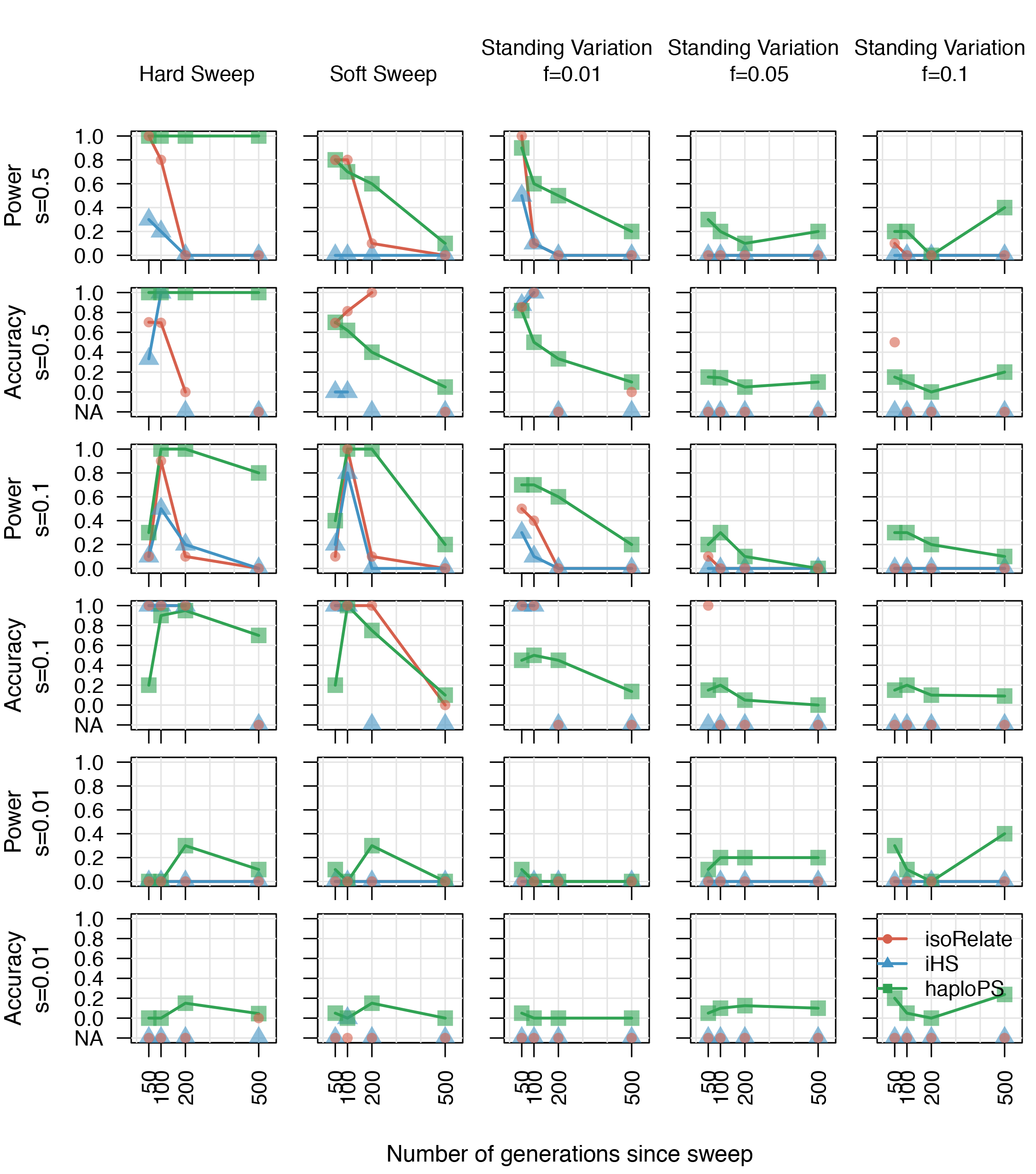

Supplement: S4 Fig — Power is defined as the proportion of sweeps (calculated over 10 reps) with at least one 20 kb bin within 50 kb either side of the selected SNP that either contains three or more significant SNPs (isoRelate and iHS, alpha = 5%), or is in the top 1% of bins with respect to the average number of haplotype counts per bin (haploPS), as a function of the number of generations since the sweep was introduced. Accuracy is calculated as either the proportion of 20 kb bins with at least three significant SNPs (isoRelate and iHS) that are within 50kb of the selected SNP or the proportion of 20 kb bins within the top 1% of bins with respect to of haplotype counts (haploPS), that are within 50kb of the selected SNP, as a function of the number of generations since the sweep was introduced. If there are no bins with at least three significant SNPs for any of the 10 reps then the accuracy is set to NA. Power values close to one indicate that most sweeps were detected. Accuracy values close to one indicate that, of the bins with 3 or more significant SNPs (or in the top 1% of bins w.r.t. haplotype counts), most were within 50kb of the sweep. The parameter s is the selection coefficient, while f is the frequency of the allele when it became positively selected. (TIF) [file pgen.1007279.s004.tif]

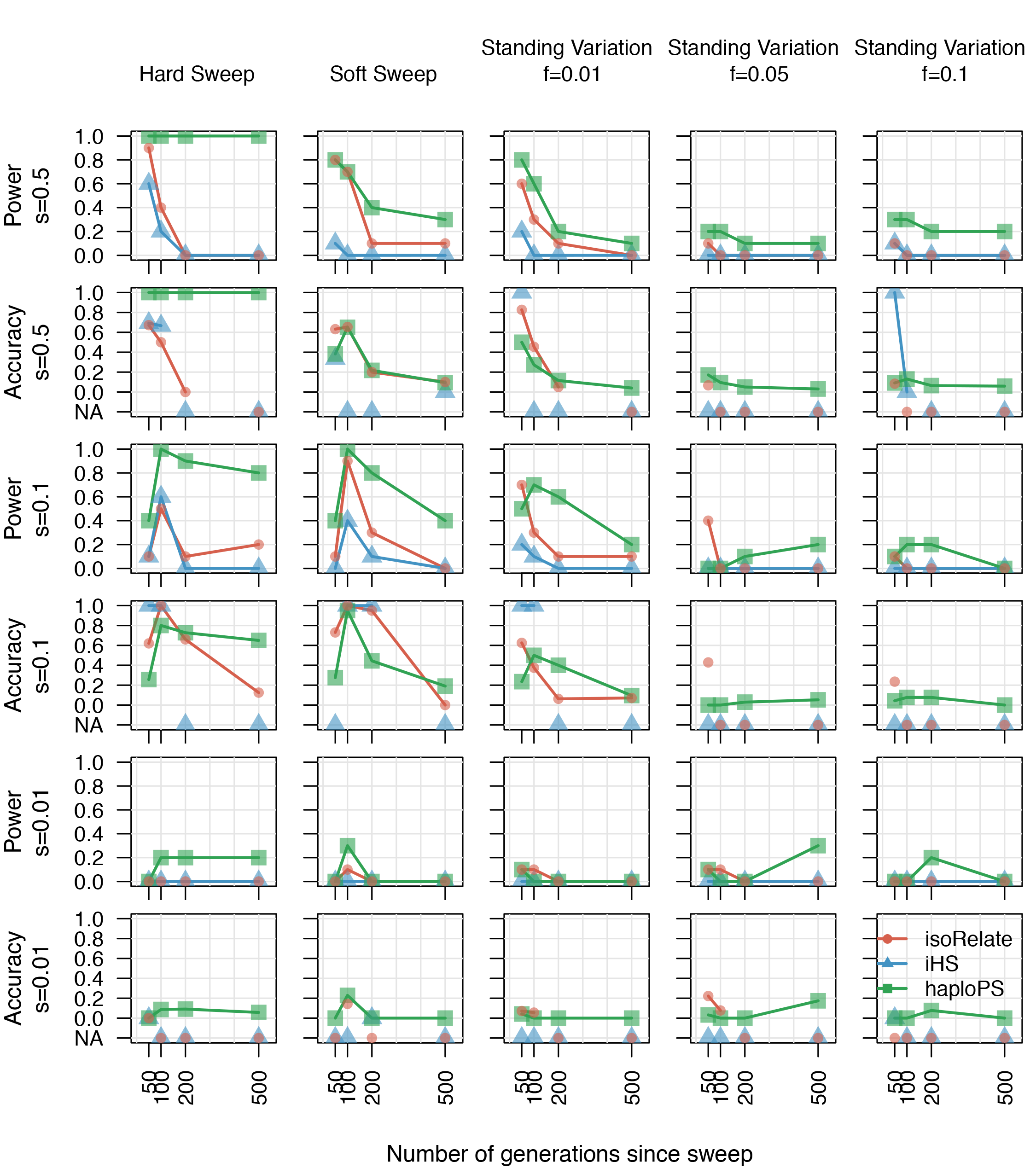

Supplement: S5 Fig — isoRelate was run on all isolates while iHS and haploPS were run on MOI = 1 isolates only. (TIF) [file pgen.1007279.s005.tif]

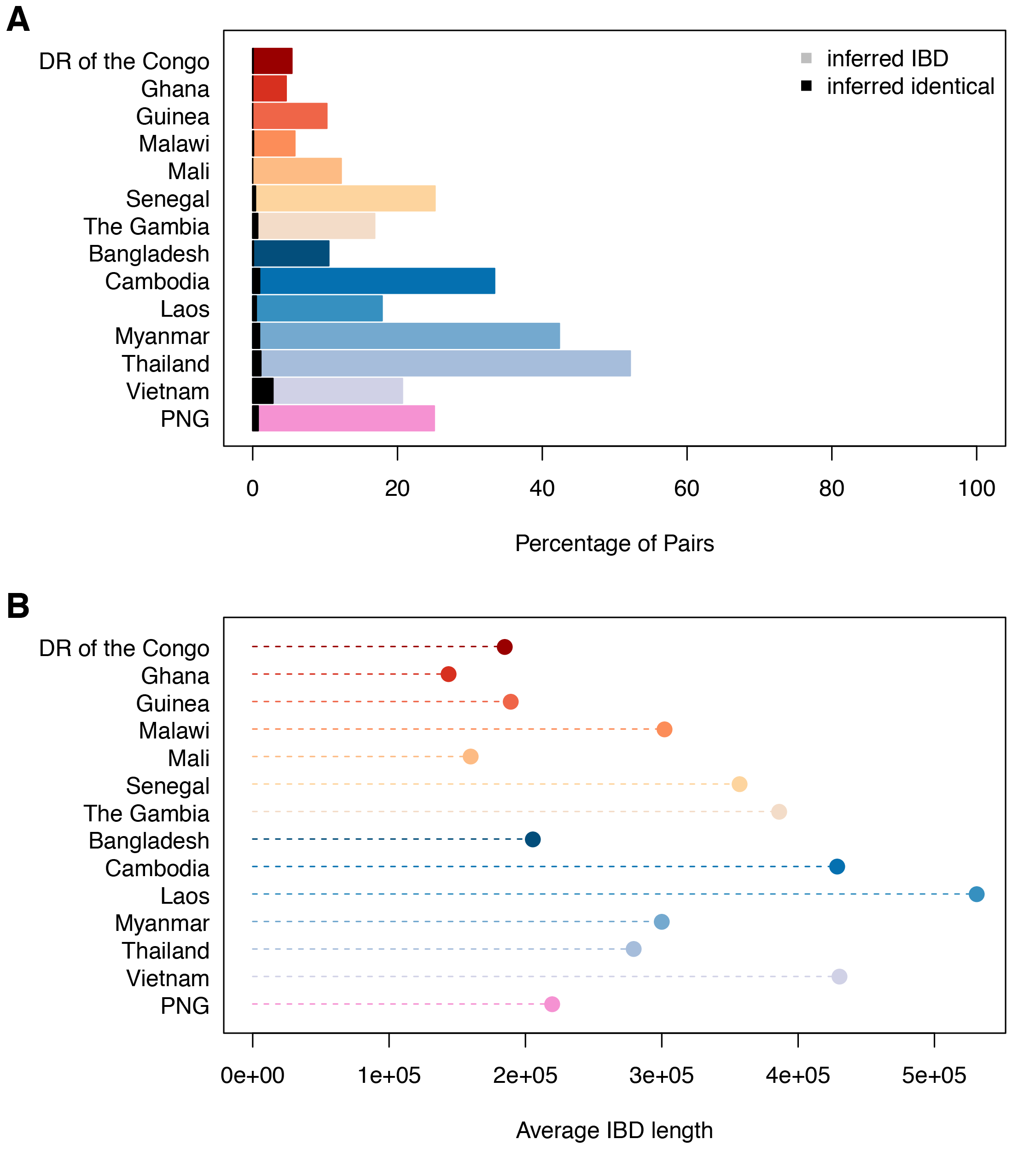

Supplement: S6 Fig — (A) The percentage of pairs with any inferred IBD at least 50 kb in length within each country and the percentage of pairs with identical genomes. (B) The average IBD length in base-pairs. (TIF) [file pgen.1007279.s006.tif]

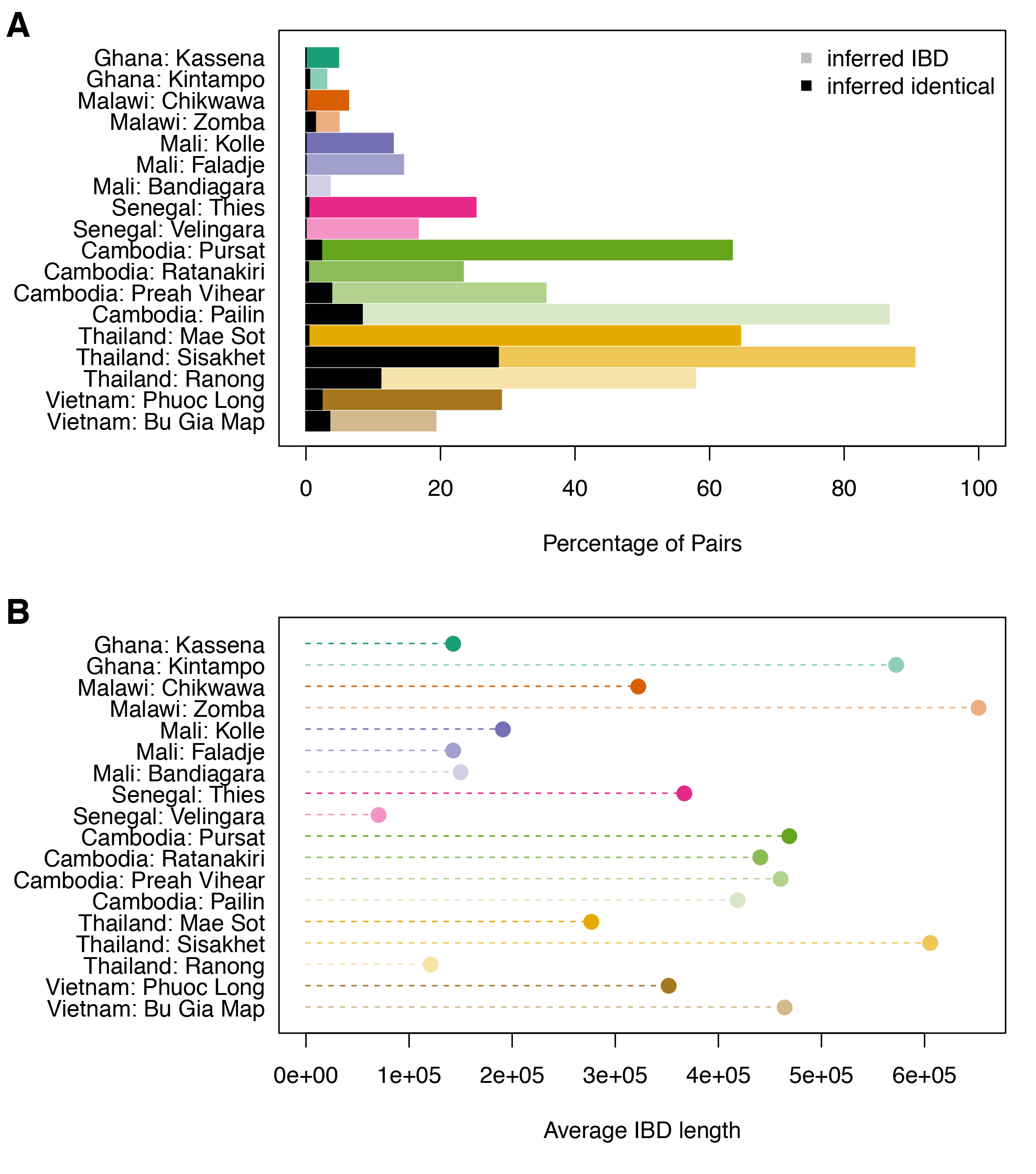

Supplement: S7 Fig — (A) The percentage of pairs with any inferred IBD at least 50 kb in length within each site and the percentage of pairs with identical genomes. (B) The average IBD length in base-pairs. (TIF) [file pgen.1007279.s007.tif]

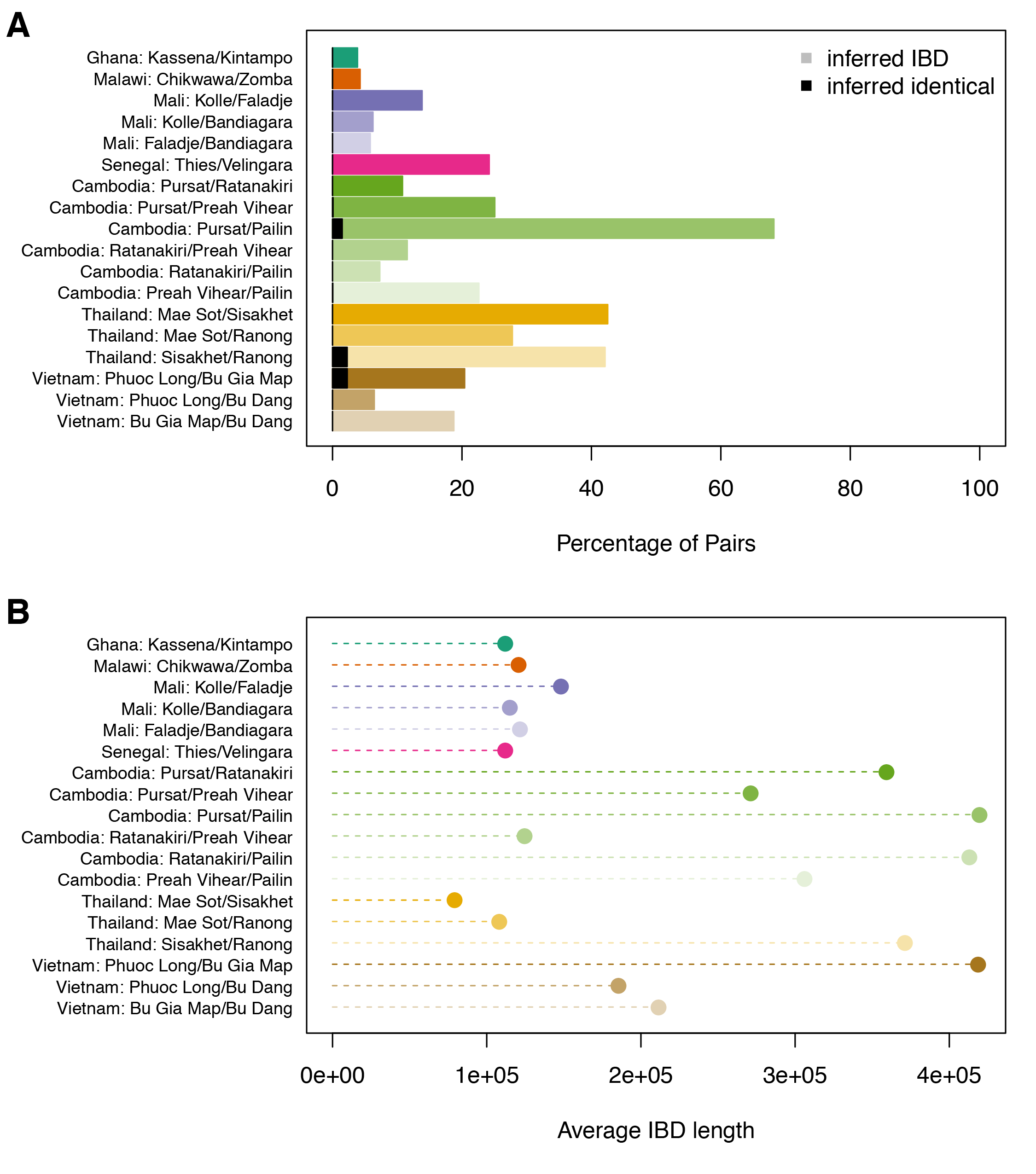

Supplement: S8 Fig — (A) The percentage of pairs with any inferred IBD at least 50 kb in length between sites within a country and the percentage of pairs with identical genomes. (B) The average IBD length in base-pairs. (TIF) [file pgen.1007279.s008.tif]

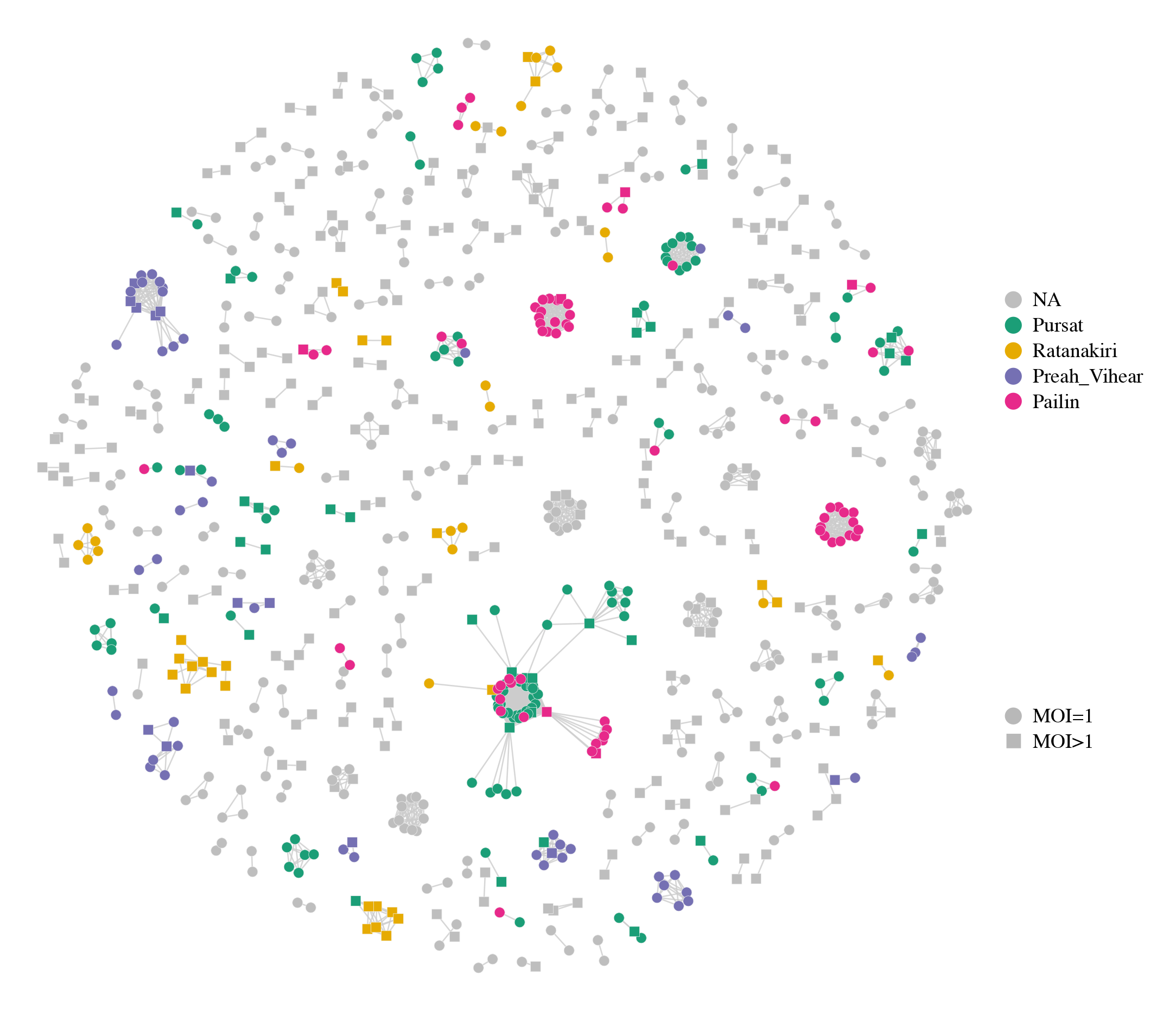

Supplement: S9 Fig — Each node identifies a unique isolate and an edge is drawn between two isolates if they share more than 90% of their genome IBD. Isolates with MOI = 1 are represented by circles while isolates with MOI > 1 are represented by squares. Only isolates collected from sites in Cambodia have a non-grey colour, with all other isolates coloured grey (i.e. isolates from all other countries). (TIF) [file pgen.1007279.s009.tif]

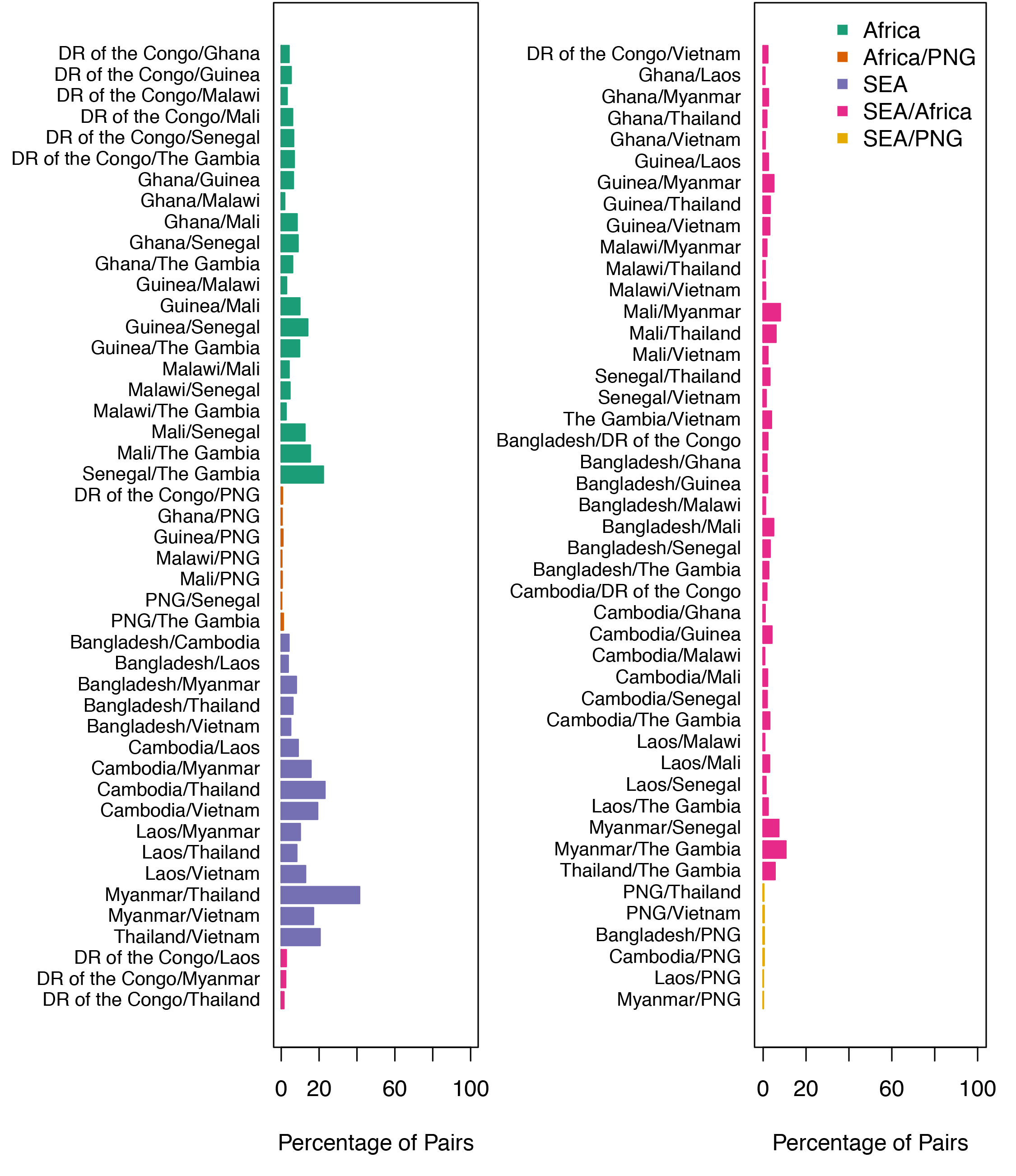

Supplement: S10 Fig — (TIF) [file pgen.1007279.s010.tif]

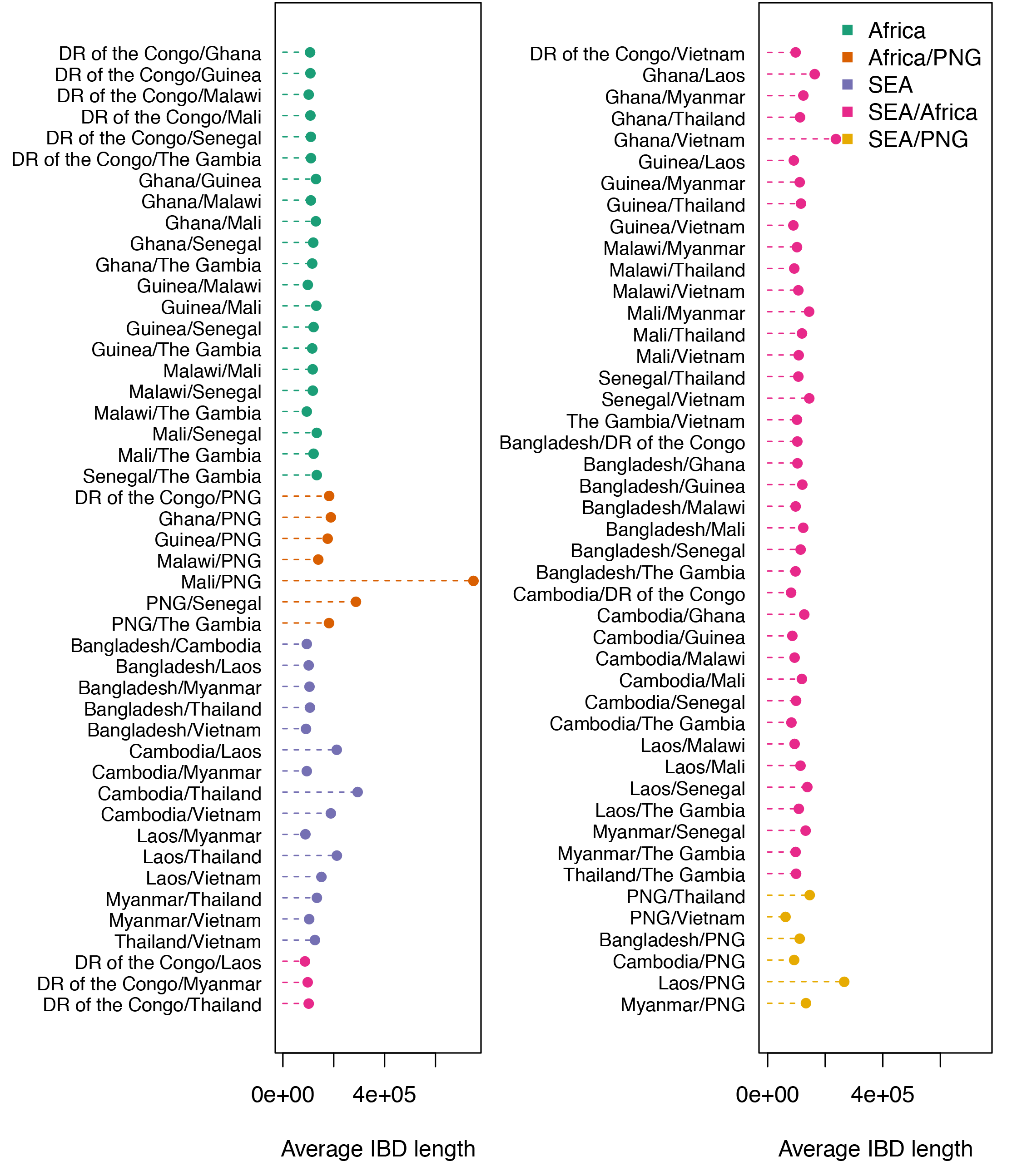

Supplement: S11 Fig — (TIF) [file pgen.1007279.s011.tif]

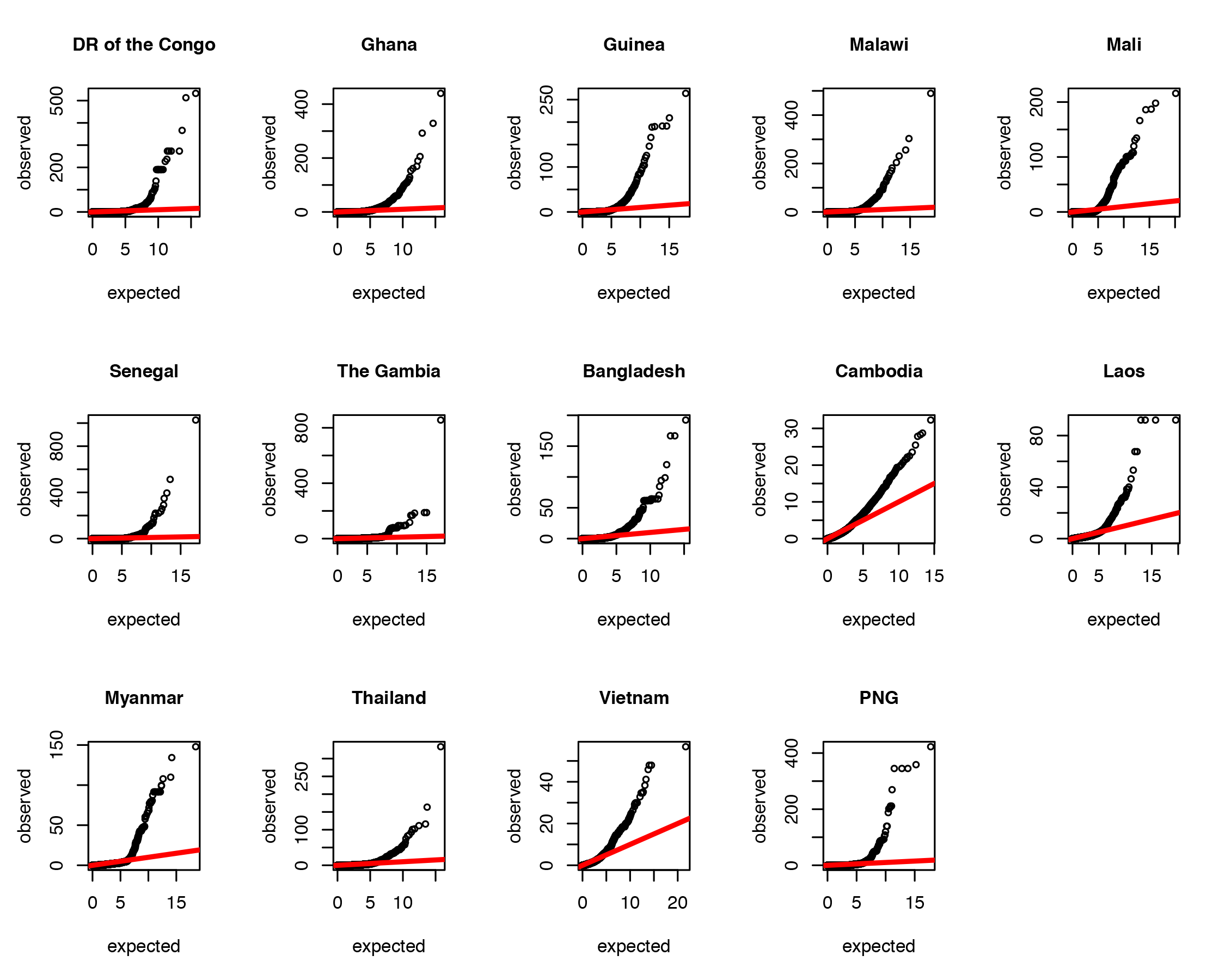

Supplement: S12 Fig — The summary statistic was calculated over all MOI ≥ 1. (TIF) [file pgen.1007279.s012.tif]
