# Supplementary material for: Identity-by-descent analyses for measuring population dynamics and selection in recombining pathogens
Source: PLoS Genet. 2018 May 23;14(5):e1007279. doi: 10.1371/journal.pgen.1007279 (PMC5988311; doi:10.1371/journal.pgen.1007279)
Supplement: S1 Table — (DOCX) [file pgen.1007279.s013.docx]

**S1 Table. The number of isolates and SNPs before and after filtering procedures for the *P. falciparum* genetic cross dataset**.

|  | **Pre VCF filtering** | | **Post VCF filtering** | | **Post isoRelate filtering** | |
| --- | --- | --- | --- | --- | --- | --- |
| **Cross** | **No. isolates** | **No. SNPs** | **No. isolates** | **No. SNPs** | **No. isolates** | **No. SNPs** |
| 3D7 x HB3 | 21 | 15,398 | 21 | 11,612 | 21 | 11,612 |
| 7G8 x GB4 | 40 | 14,426 | 40 | 10,903 | 40 | 10,903 |
| HB3 x Dd2 | 37 | 14,914 | 37 | 10,637 | 37 | 10,637 |
