# Supplementary material for: Identity-by-descent analyses for measuring population dynamics and selection in recombining pathogens
Source: PLoS Genet. 2018 May 23;14(5):e1007279. doi: 10.1371/journal.pgen.1007279 (PMC5988311; doi:10.1371/journal.pgen.1007279)
Supplement: S2 Table — The percentages were averaged over all 150 datasets corresponding to the parameter combinations assessed. (DOCX) [file pgen.1007279.s014.docx]

**S2 Table. The average percentage of isolates with various simulated MOI.** The percentages were averaged over all 150 datasets corresponding to the parameter combinations assessed.

| **MOI** | | | | | | |
| --- | --- | --- | --- | --- | --- | --- |
| **1** | **2** | **3** | **4** | **5** | **6** | **7** |
| 56.81% | 30.03% | 10.01% | 2.55% | 0.48% | 0.11% | 0.01% |
