# Supplementary material for: Identity-by-descent analyses for measuring population dynamics and selection in recombining pathogens
Source: PLoS Genet. 2018 May 23;14(5):e1007279. doi: 10.1371/journal.pgen.1007279 (PMC5988311; doi:10.1371/journal.pgen.1007279)
Supplement: S3 Table — (DOCX) [file pgen.1007279.s015.docx]

**S3 Table. The sample collection years and the number of isolates and SNPs after filtering procedures within each country.**

|  |  |  |  | **Post VCF filtering** | | **Post isoRelate filtering** | |
| --- | --- | --- | --- | --- | --- | --- | --- |
| **Region** | **Country** | **Collection year (min)^a^** | **Collection Year (max)^a^** | **No. isolates** | **No. SNPs** | **No. isolates** | **No. SNPs** |
| Africa | DR of the Congo | 2013 | 2013 | 104 | 60,969 | 104 | 31,676 |
| Africa | Ghana | 2009 | 2013 | 563 | 258,289 | 563 | 28,483 |
| Africa | Guinea | 2011 | 2011 | 100 | 101,425 | 100 | 44,528 |
| Africa | Malawi | 2011 | 2011 | 357 | 154,265 | 357 | 40,225 |
| Africa | Mali | 2007 | 2007 | 84 | 46,668 | 84 | 19,339 |
| Africa | Senegal | 2001 | 2011 | 131 | 52,664 | 131 | 26,757 |
| Africa | The Gambia | 2008 | 2008 | 57 | 46,576 | 57 | 43,360 |
| Southeast Asia | Bangladesh | 2012 | 2012 | 45 | 34,708 | 45 | 32,322 |
| Southeast Asia | Cambodia | 2009 | 2012 | 521 | 58,987 | 521 | 28,448 |
| Southeast Asia | Laos | 2011 | 2012 | 84 | 46,447 | 84 | 33,006 |
| Southeast Asia | Myanmar | 2011 | 2013 | 57 | 33,179 | 57 | 29,997 |
| Southeast Asia | Thailand | 2011 | 2013 | 140 | 40,502 | 140 | 28,218 |
| Southeast Asia | Vietnam | 2011 | 2012 | 96 | 42,011 | 96 | 29,617 |
| Oceania | PNG^b^ | 2007 | 2007 | 38 | 29,631 | 37 | 18,270 |

^a^ Minimum and maximum collection years were obtained from pf3k metadata.

^b^ Papua New Guinea is represented by the acronym PNG.
