# Supplementary material for: Identity-by-descent analyses for measuring population dynamics and selection in recombining pathogens
Source: PLoS Genet. 2018 May 23;14(5):e1007279. doi: 10.1371/journal.pgen.1007279 (PMC5988311; doi:10.1371/journal.pgen.1007279)
Supplement: S4 Table — (DOCX) [file pgen.1007279.s016.docx]

**S4 Table. The number and percentage of isolates with MOI = 1 and MOI > 1 within each country.**

| **Region** | **Country** | **MOI = 1** | **MO1 > 1** |
| --- | --- | --- | --- |
| Africa | DR of the Congo | 47 (45%) | 57 (55%) |
| Africa | Ghana | 243 (43%) | 320 (57%) |
| Africa | Guinea | 51 (51%) | 49 (49%) |
| Africa | Malawi | 134 (37%) | 223 (63%) |
| Africa | Mali | 40 (48%) | 44 (52%) |
| Africa | Senegal | 110 (84%) | 21 (16%) |
| Africa | The Gambia | 40 (70%) | 17 (30%) |
| Southeast Asia | Bangladesh | 21 (47%) | 24 (53%) |
| Southeast Asia | Cambodia | 396 (76%) | 125 (24%) |
| Southeast Asia | Laos | 49 (58%) | 35 (42%) |
| Southeast Asia | Myanmar | 45 (79%) | 12 (21%) |
| Southeast Asia | Thailand | 107 (76%) | 33 (24%) |
| Southeast Asia | Vietnam | 69 (72%) | 27 (28%) |
| Oceania | PNG | 31 (82%) | 7 (18%) |
