# Supplementary material for: Identity-by-descent analyses for measuring population dynamics and selection in recombining pathogens
Source: PLoS Genet. 2018 May 23;14(5):e1007279. doi: 10.1371/journal.pgen.1007279 (PMC5988311; doi:10.1371/journal.pgen.1007279)
Supplement: S5 Table — (DOCX) [file pgen.1007279.s017.docx]

**S5 Table. The number of isolates and SNPs included in the IBD analyses between pairs of countries.**

| **Region A** | **Region B** | **Country A** | **Country B** | **No. isolates** | **No. SNPs** |
| --- | --- | --- | --- | --- | --- |
| Africa | Africa | DR of the Congo | Ghana | 667 | 19,167 |
| Africa | Africa | DR of the Congo | Guinea | 204 | 19,236 |
| Africa | Africa | DR of the Congo | Malawi | 461 | 20,110 |
| Africa | Africa | DR of the Congo | Mali | 188 | 16,439 |
| Africa | Africa | DR of the Congo | Senegal | 235 | 16,529 |
| Africa | Africa | DR of the Congo | The Gambia | 161 | 16,192 |
| Africa | Southeast Asia | DR of the Congo | Bangladesh | 149 | 10,970 |
| Africa | Southeast Asia | DR of the Congo | Cambodia | 625 | 7,775 |
| Africa | Southeast Asia | DR of the Congo | Laos | 188 | 7,814 |
| Africa | Southeast Asia | DR of the Congo | Myanmar | 161 | 7,211 |
| Africa | Southeast Asia | DR of the Congo | Thailand | 244 | 7,353 |
| Africa | Southeast Asia | DR of the Congo | Vietnam | 200 | 7,390 |
| Africa | Oceania | DR of the Congo | PNG | 142 | 2,813 |
| Africa | Africa | Ghana | Guinea | 663 | 26,656 |
| Africa | Africa | Ghana | Malawi | 920 | 26,595 |
| Africa | Africa | Ghana | Mali | 647 | 16,127 |
| Africa | Africa | Ghana | Senegal | 694 | 18,138 |
| Africa | Africa | Ghana | The Gambia | 620 | 19,478 |
| Africa | Southeast Asia | Ghana | Bangladesh | 608 | 11,689 |
| Africa | Southeast Asia | Ghana | Cambodia | 1084 | 12,457 |
| Africa | Southeast Asia | Ghana | Laos | 647 | 10,334 |
| Africa | Southeast Asia | Ghana | Myanmar | 620 | 9,398 |
| Africa | Southeast Asia | Ghana | Thailand | 703 | 10,680 |
| Africa | Southeast Asia | Ghana | Vietnam | 659 | 10,046 |
| Africa | Oceania | Ghana | PNG | 600 | 4,118 |
| Africa | Africa | Guinea | Malawi | 457 | 29,138 |
| Africa | Africa | Guinea | Mali | 184 | 18,079 |
| Africa | Africa | Guinea | Senegal | 231 | 18,497 |
| Africa | Africa | Guinea | The Gambia | 157 | 21,457 |
| Africa | Southeast Asia | Guinea | Bangladesh | 145 | 11,937 |
| Africa | Southeast Asia | Guinea | Cambodia | 621 | 10,270 |
| Africa | Southeast Asia | Guinea | Laos | 184 | 11,458 |
| Africa | Southeast Asia | Guinea | Myanmar | 157 | 10,129 |
| Africa | Southeast Asia | Guinea | Thailand | 240 | 10,428 |
| Africa | Southeast Asia | Guinea | Vietnam | 196 | 10,756 |
| Africa | Oceania | Guinea | PNG | 134 | 4,834 |
| Africa | Africa | Malawi | Mali | 441 | 14,493 |
| Africa | Africa | Malawi | Senegal | 488 | 16,373 |
| Africa | Africa | Malawi | The Gambia | 414 | 19,049 |
| Africa | Southeast Asia | Malawi | Bangladesh | 402 | 12,405 |
| Africa | Southeast Asia | Malawi | Cambodia | 878 | 12,233 |
| Africa | Southeast Asia | Malawi | Laos | 441 | 12,638 |
| Africa | Southeast Asia | Malawi | Myanmar | 414 | 10,837 |
| Africa | Southeast Asia | Malawi | Thailand | 497 | 11,657 |
| Africa | Southeast Asia | Malawi | Vietnam | 453 | 11,591 |
| Africa | Oceania | Malawi | PNG | 391 | 4,988 |
| Africa | Africa | Mali | Senegal | 215 | 17,458 |
| Africa | Africa | Mali | The Gambia | 141 | 13,899 |
| Africa | Southeast Asia | Mali | Bangladesh | 129 | 8,997 |
| Africa | Southeast Asia | Mali | Cambodia | 605 | 5,941 |
| Africa | Southeast Asia | Mali | Laos | 168 | 5,805 |
| Africa | Southeast Asia | Mali | Myanmar | 141 | 5,622 |
| Africa | Southeast Asia | Mali | Thailand | 224 | 5,507 |
| Africa | Southeast Asia | Mali | Vietnam | 180 | 5,555 |
| Africa | Oceania | Mali | PNG | 122 | 1,945 |
| Africa | Africa | Senegal | The Gambia | 188 | 16,364 |
| Africa | Southeast Asia | Senegal | Bangladesh | 176 | 9,275 |
| Africa | Southeast Asia | Senegal | Cambodia | 652 | 6,486 |
| Africa | Southeast Asia | Senegal | Laos | 215 | 6,315 |
| Africa | Southeast Asia | Senegal | Myanmar | 188 | 5,885 |
| Africa | Southeast Asia | Senegal | Thailand | 271 | 6,017 |
| Africa | Southeast Asia | Senegal | Vietnam | 227 | 6,011 |
| Africa | Oceania | Senegal | PNG | 169 | 2,274 |
| Africa | Southeast Asia | The Gambia | Bangladesh | 102 | 9,882 |
| Africa | Southeast Asia | The Gambia | Cambodia | 578 | 7,305 |
| Africa | Southeast Asia | The Gambia | Laos | 141 | 7,979 |
| Africa | Southeast Asia | The Gambia | Myanmar | 114 | 7,305 |
| Africa | Southeast Asia | The Gambia | Thailand | 197 | 7,398 |
| Africa | Southeast Asia | The Gambia | Vietnam | 153 | 7,504 |
| Africa | Oceania | The Gambia | PNG | 94 | 2,984 |
| Southeast Asia | Southeast Asia | Bangladesh | Cambodia | 566 | 13,736 |
| Southeast Asia | Southeast Asia | Bangladesh | Laos | 129 | 15,029 |
| Southeast Asia | Southeast Asia | Bangladesh | Myanmar | 102 | 14,751 |
| Southeast Asia | Southeast Asia | Bangladesh | Thailand | 185 | 14,666 |
| Southeast Asia | Southeast Asia | Bangladesh | Vietnam | 141 | 14,207 |
| Southeast Asia | Oceania | Bangladesh | PNG | 83 | 4,218 |
| Southeast Asia | Southeast Asia | Cambodia | Laos | 605 | 24,921 |
| Southeast Asia | Southeast Asia | Cambodia | Myanmar | 578 | 17,479 |
| Southeast Asia | Southeast Asia | Cambodia | Thailand | 661 | 21,199 |
| Southeast Asia | Southeast Asia | Cambodia | Vietnam | 617 | 23,394 |
| Southeast Asia | Oceania | Cambodia | PNG | 558 | 5,395 |
| Southeast Asia | Southeast Asia | Laos | Myanmar | 141 | 19,045 |
| Southeast Asia | Southeast Asia | Laos | Thailand | 224 | 21,164 |
| Southeast Asia | Southeast Asia | Laos | Vietnam | 180 | 26,419 |
| Southeast Asia | Oceania | Laos | PNG | 121 | 5,994 |
| Southeast Asia | Southeast Asia | Myanmar | Thailand | 197 | 21,293 |
| Southeast Asia | Southeast Asia | Myanmar | Vietnam | 153 | 18,666 |
| Southeast Asia | Oceania | Myanmar | PNG | 94 | 5,124 |
| Southeast Asia | Southeast Asia | Thailand | Vietnam | 236 | 21,069 |
| Southeast Asia | Oceania | Thailand | PNG | 177 | 5,572 |
| Southeast Asia | Oceania | Vietnam | PNG | 133 | 5,734 |
