# Supplementary material for: Identity-by-descent analyses for measuring population dynamics and selection in recombining pathogens
Source: PLoS Genet. 2018 May 23;14(5):e1007279. doi: 10.1371/journal.pgen.1007279 (PMC5988311; doi:10.1371/journal.pgen.1007279)
Supplement: S6 Table — (DOCX) [file pgen.1007279.s018.docx]

**S6 Table. Summary of relatedness between pairs of isolates within the same country.**

| **Region** | **Country** | **No. isolates** | **No. pairs** | **% of pairs IBD^a^** | **% of pairs identical^b^** | **Ave. % of pairs IBD per SNP^c^** | **Ave. % of genome IBD^d^** | **Ave. length of IBD (kb)^e^** |
| --- | --- | --- | --- | --- | --- | --- | --- | --- |
| Africa | DR of the Congo | 104 | 5,356 | 5.41 | 0.06 | 0.12 | 1.06 | 185 |
| Africa | Ghana | 563 | 158,203 | 4.62 | 0.01 | 0.06 | 0.78 | 144 |
| Africa | Guinea | 100 | 4,950 | 10.24 | 0 | 0.16 | 1.46 | 189 |
| Africa | Malawi | 357 | 63,546 | 5.82 | 0.11 | 0.24 | 2.24 | 302 |
| Africa | Mali | 84 | 3,486 | 12.22 | 0 | 0.15 | 0.83 | 160 |
| Africa | Senegal | 131 | 8,515 | 25.18 | 0.38 | 1.13 | 2.94 | 357 |
| Africa | The Gambia | 57 | 1,596 | 16.85 | 0.69 | 1.59 | 5.44 | 386 |
| Southeast Asia | Bangladesh | 45 | 990 | 10.51 | 0.1 | 0.27 | 1.3 | 205 |
| Southeast Asia | Cambodia | 521 | 135,460 | 33.41 | 0.95 | 5.38 | 13.72 | 429 |
| Southeast Asia | Laos | 84 | 3,486 | 17.87 | 0.49 | 2.06 | 8.86 | 531 |
| Southeast Asia | Myanmar | 57 | 1,596 | 42.36 | 0.94 | 2.6 | 3.82 | 300 |
| Southeast Asia | Thailand | 140 | 97,30 | 52.15 | 1.12 | 3.18 | 3.8 | 280 |
| Southeast Asia | Vietnam | 96 | 4,560 | 20.68 | 2.79 | 4.31 | 8.25 | 431 |
| Oceania | PNG | 37 | 666 | 25.08 | 0.75 | 1.19 | 1.68 | 220 |

**^a^** Percentage of all pairs inferred IBD at any genomic location.

**^b^** Percentage of all pairs with identical genomes.

**^c^** Average percentage of pairs IBD calculated genome-wide.

**^d^** Average percentage of genome IBD calculated from IBD pairs only, excluding identical pairs.

**^e^** Average length of inferred IBD segments (kb), excluding segments from identical pair
