# Supplementary material for: Identity-by-descent analyses for measuring population dynamics and selection in recombining pathogens
Source: PLoS Genet. 2018 May 23;14(5):e1007279. doi: 10.1371/journal.pgen.1007279 (PMC5988311; doi:10.1371/journal.pgen.1007279)
Supplement: S7 Table — Only countries with multiple study sites are displayed. (DOCX) [file pgen.1007279.s019.docx]

**S7 Table**. **Summary of relatedness between pairs of isolates within the same country, stratified by study location.** Only countries with multiple study sites are displayed.

| **Region** | **Country** | **Site** | **No. isolates** | **No. pairs** | **% of pairs IBD** | **% of pairs identical** | **Ave. % of pairs IBD per SNP** | **Ave. % of genome IBD** | **Ave. length of IBD (kb)** |
| --- | --- | --- | --- | --- | --- | --- | --- | --- | --- |
| Africa | Ghana | Kassena | 501 | 125,250 | 4.83 | 0.01 | 0.06 | 0.78 | 143 |
| Africa | Ghana | Kintampo | 62 | 1,891 | 3.07 | 0.53 | 0.68 | 6.04 | 572 |
| Africa | Malawi | Chikwawa | 310 | 47,895 | 6.31 | 0.11 | 0.27 | 2.35 | 322 |
| Africa | Malawi | Zomba | 47 | 1,081 | 4.9 | 1.39 | 1.99 | 16.89 | 653 |
| Africa | Mali | Kolle | 46 | 1,035 | 12.95 | 0 | 0.18 | 1.09 | 191 |
| Africa | Mali | Faladje | 30 | 435 | 14.48 | 0 | 0.17 | 0.7 | 143 |
| Africa | Mali | Bandiagara | 8 | 28 | 3.57 | 0 | 0.03 | 0.71 | 150 |
| Africa | Senegal | Thies | 127 | 8,001 | 25.25 | 0.4 | 1.19 | 3.09 | 367 |
| Africa | Senegal | Velingara | 4 | 6 | 16.67 | 0 | 0.06 | 0.34 | 71 |
| Southeast Asia | Cambodia | Pursat | 219 | 23,871 | 63.35 | 2.3 | 12.48 | 16.54 | 469 |
| Southeast Asia | Cambodia | Ratanakiri | 134 | 8,911 | 23.36 | 0.36 | 1.97 | 6.74 | 441 |
| Southeast Asia | Cambodia | Preah Vihear | 86 | 3,655 | 35.65 | 3.8 | 7.14 | 10.3 | 461 |
| Southeast Asia | Cambodia | Pailin | 82 | 3,321 | 86.66 | 8.34 | 29.08 | 27.18 | 419 |
| Southeast Asia | Thailand | Mae Sot | 100 | 4,950 | 64.57 | 0.42 | 3.46 | 4.48 | 277 |
| Southeast Asia | Thailand | Sisakhet | 21 | 210 | 90.48 | 28.57 | 43.84 | 25.06 | 606 |
| Southeast Asia | Thailand | Ranong | 19 | 171 | 57.89 | 11.11 | 11.83 | 1.17 | 121 |
| Southeast Asia | Vietnam | Phuoc Long | 31 | 465 | 29.03 | 2.37 | 3.71 | 4.74 | 352 |
| Southeast Asia | Vietnam | Bu Gia Map | 64 | 2,016 | 19.3 | 3.52 | 5.22 | 10.5 | 464 |
