# Supplementary material for: Identity-by-descent analyses for measuring population dynamics and selection in recombining pathogens
Source: PLoS Genet. 2018 May 23;14(5):e1007279. doi: 10.1371/journal.pgen.1007279 (PMC5988311; doi:10.1371/journal.pgen.1007279)
Supplement: S8 Table — (DOCX) [file pgen.1007279.s020.docx]

**S8 Table. Summary of relatedness between pairs of isolates from different sites within a country.**

| **Region** | **Country** | **Site A** | **Site B** | **No. isolates** | **No. pairs** | **% of pairs IBD** | **% of pairs identical** | **Ave. % of pairs IBD per SNP** | **Ave. % of genome IBD** | **Ave. length of IBD (kb)** |
| --- | --- | --- | --- | --- | --- | --- | --- | --- | --- | --- |
| Africa | Ghana | Kassena | Kintampo | 563 | 31,062 | 3.86 | 0 | 0.03 | 0.55 | 112 |
| Africa | Malawi | Chikwawa | Zomba | 357 | 14,570 | 4.26 | 0 | 0.04 | 0.84 | 121 |
| Africa | Mali | Kolle | Faladje | 76 | 1,380 | 13.84 | 0 | 0.16 | 0.74 | 148 |
| Africa | Mali | Kolle | Bandiagara | 54 | 368 | 6.25 | 0 | 0.05 | 0.57 | 115 |
| Africa | Mali | Faladje | Bandiagara | 38 | 240 | 5.83 | 0 | 0.04 | 0.58 | 122 |
| Africa | Senegal | Thies | Velingara | 131 | 508 | 24.21 | 0 | 0.17 | 0.6 | 112 |
| Southeast Asia | Cambodia | Pursat | Ratanakiri | 353 | 29,346 | 10.81 | 0 | 0.29 | 2.52 | 359 |
| Southeast Asia | Cambodia | Pursat | Preah Vihear | 305 | 18,834 | 25.07 | 0.07 | 0.89 | 3.04 | 271 |
| Southeast Asia | Cambodia | Pursat | Pailin | 301 | 17,958 | 68.2 | 1.49 | 14.08 | 19.41 | 420 |
| Southeast Asia | Cambodia | Ratanakiri | Preah Vihear | 220 | 11,524 | 11.54 | 0 | 0.11 | 0.71 | 125 |
| Southeast Asia | Cambodia | Ratanakiri | Pailin | 216 | 10,988 | 7.3 | 0 | 0.25 | 3.35 | 413 |
| Southeast Asia | Cambodia | Preah Vihear | Pailin | 168 | 7,052 | 22.62 | 0.04 | 1.12 | 4.44 | 306 |
| Southeast Asia | Thailand | Mae Sot | Sisakhet | 121 | 2,100 | 42.52 | 0 | 0.29 | 0.48 | 79 |
| Southeast Asia | Thailand | Mae Sot | Ranong | 119 | 1,900 | 27.79 | 0 | 0.26 | 0.65 | 108 |
| Southeast Asia | Thailand | Sisakhet | Ranong | 40 | 399 | 42.11 | 2.26 | 3.65 | 3.43 | 371 |
| Southeast Asia | Vietnam | Phuoc Long | Bu Gia Map | 95 | 1,984 | 20.41 | 2.27 | 3.72 | 7.7 | 419 |
| Southeast Asia | Vietnam | Phuoc Long | Bu Dang | 32 | 31 | 6.45 | 0 | 0.06 | 0.88 | 186 |
| Southeast Asia | Vietnam | Bu Gia Map | Bu Dang | 65 | 64 | 18.75 | 0 | 0.54 | 2.59 | 212 |
