# Supplementary material for: Identity-by-descent analyses for measuring population dynamics and selection in recombining pathogens
Source: PLoS Genet. 2018 May 23;14(5):e1007279. doi: 10.1371/journal.pgen.1007279 (PMC5988311; doi:10.1371/journal.pgen.1007279)
Supplement: S9 Table — (DOCX) [file pgen.1007279.s021.docx]

**S9 Table**. **Summary of relatedness between pairs of isolates from different countries.**

| **Region A** | **Region B** | **Country A** | **Country B** | **No. isolates** | **No. pairs** | **% of pairs IBD** | **% of pairs identical** | **Ave. % of pairs IBD per SNP** | **Ave. % of genome IBD** | **Ave. length of IBD (kb)** |
| --- | --- | --- | --- | --- | --- | --- | --- | --- | --- | --- |
| Africa | Africa | DR of the Congo | Ghana | 667 | 58,552 | 4.12 | 0 | 0.03 | 0.52 | 108 |
| Africa | Africa | DR of the Congo | Guinea | 204 | 10,400 | 5.25 | 0 | 0.04 | 0.52 | 109 |
| Africa | Africa | DR of the Congo | Malawi | 461 | 37,128 | 3.1 | 0 | 0.02 | 0.49 | 102 |
| Africa | Africa | DR of the Congo | Mali | 188 | 8,736 | 5.99 | 0 | 0.04 | 0.52 | 108 |
| Africa | Africa | DR of the Congo | Senegal | 235 | 13,624 | 6.55 | 0 | 0.04 | 0.54 | 110 |
| Africa | Africa | DR of the Congo | The Gambia | 161 | 5,928 | 6.87 | 0 | 0.05 | 0.53 | 111 |
| Africa | Africa | Ghana | Guinea | 663 | 56,300 | 6.41 | 0 | 0.07 | 0.79 | 131 |
| Africa | Africa | Ghana | Malawi | 920 | 200,991 | 1.83 | 0 | 0.01 | 0.58 | 110 |
| Africa | Africa | Ghana | Mali | 647 | 47,292 | 8.37 | 0 | 0.09 | 0.64 | 130 |
| Africa | Africa | Ghana | Senegal | 694 | 73,753 | 8.88 | 0 | 0.07 | 0.62 | 120 |
| Africa | Africa | Ghana | The Gambia | 620 | 32,091 | 6.01 | 0 | 0.06 | 0.57 | 115 |
| Africa | Africa | Guinea | Malawi | 457 | 35,700 | 2.81 | 0 | 0.02 | 0.63 | 98 |
| Africa | Africa | Guinea | Mali | 184 | 8,400 | 9.83 | 0 | 0.09 | 0.65 | 132 |
| Africa | Africa | Guinea | Senegal | 231 | 13,100 | 13.98 | 0 | 0.12 | 0.65 | 120 |
| Africa | Africa | Guinea | The Gambia | 157 | 5,700 | 9.67 | 0 | 0.09 | 0.6 | 115 |
| Africa | Africa | Malawi | Mali | 441 | 29,988 | 4.16 | 0 | 0.03 | 0.56 | 117 |
| Africa | Africa | Malawi | Senegal | 488 | 46,767 | 4.6 | 0 | 0.03 | 0.59 | 119 |
| Africa | Africa | Malawi | The Gambia | 414 | 20,349 | 2.55 | 0 | 0.02 | 0.45 | 94 |
| Africa | Africa | Mali | Senegal | 215 | 11,004 | 12.5 | 0 | 0.12 | 0.67 | 133 |
| Africa | Africa | Mali | The Gambia | 141 | 4,788 | 15.35 | 0 | 0.17 | 0.61 | 121 |
| Africa | Africa | Senegal | The Gambia | 188 | 7,467 | 22.24 | 0 | 0.24 | 0.77 | 133 |
| Africa | Southeast Asia | DR of the Congo | Bangladesh | 149 | 4,680 | 2.2 | 0 | 0.03 | 0.49 | 103 |
| Africa | Southeast Asia | DR of the Congo | Cambodia | 625 | 54,184 | 1.7 | 0 | 0.03 | 0.39 | 81 |
| Africa | Southeast Asia | DR of the Congo | Laos | 188 | 8,736 | 2.66 | 0 | 0.05 | 0.41 | 86 |
| Africa | Southeast Asia | DR of the Congo | Myanmar | 161 | 5,928 | 2.34 | 0 | 0.04 | 0.47 | 98 |
| Africa | Southeast Asia | DR of the Congo | Thailand | 244 | 14,560 | 1.41 | 0 | 0.03 | 0.48 | 102 |
| Africa | Southeast Asia | DR of the Congo | Vietnam | 200 | 9,984 | 2.18 | 0 | 0.04 | 0.46 | 97 |
| Africa | Southeast Asia | Ghana | Bangladesh | 608 | 25,335 | 1.75 | 0 | 0.02 | 0.49 | 103 |
| Africa | Southeast Asia | Ghana | Cambodia | 1084 | 29,3323 | 0.92 | 0 | 0.01 | 0.65 | 1285 |
| Africa | Southeast Asia | Ghana | Laos | 647 | 47,292 | 0.84 | 0 | 0.01 | 0.88 | 164 |
| Africa | Southeast Asia | Ghana | Myanmar | 620 | 32,091 | 2.44 | 0 | 0.03 | 0.61 | 124 |
| Africa | Southeast Asia | Ghana | Thailand | 703 | 78,820 | 1.7 | 0 | 0.02 | 0.56 | 113 |
| Africa | Southeast Asia | Ghana | Vietnam | 659 | 54,048 | 0.97 | 0 | 0.02 | 1.41 | 237 |
| Africa | Southeast Asia | Guinea | Bangladesh | 145 | 4,500 | 2.02 | 0 | 0.03 | 0.58 | 120 |
| Africa | Southeast Asia | Guinea | Cambodia | 621 | 52,100 | 4.07 | 0 | 0.05 | 0.41 | 86 |
| Africa | Southeast Asia | Guinea | Laos | 184 | 8,400 | 2.42 | 0 | 0.04 | 0.44 | 91 |
| Africa | Southeast Asia | Guinea | Myanmar | 157 | 5,700 | 4.98 | 0 | 0.07 | 0.55 | 112 |
| Africa | Southeast Asia | Guinea | Thailand | 240 | 14,000 | 3.3 | 0 | 0.05 | 0.56 | 116 |
| Africa | Southeast Asia | Guinea | Vietnam | 196 | 9,600 | 3.08 | 0 | 0.05 | 0.42 | 89 |
| Africa | Southeast Asia | Malawi | Bangladesh | 402 | 16,065 | 1.09 | 0 | 0.01 | 0.46 | 98 |
| Africa | Southeast Asia | Malawi | Cambodia | 878 | 185,997 | 0.71 | 0 | 0.01 | 0.45 | 94 |
| Africa | Southeast Asia | Malawi | Laos | 441 | 29,988 | 0.74 | 0 | 0.01 | 0.45 | 93 |
| Africa | Southeast Asia | Malawi | Myanmar | 414 | 20,349 | 1.72 | 0 | 0.02 | 0.49 | 103 |
| Africa | Southeast Asia | Malawi | Thailand | 497 | 49,980 | 0.98 | 0 | 0.01 | 0.44 | 93 |
| Africa | Southeast Asia | Malawi | Vietnam | 453 | 34,272 | 1.1 | 0 | 0.01 | 0.51 | 107 |
| Africa | Southeast Asia | Mali | Bangladesh | 129 | 3,780 | 4.89 | 0 | 0.06 | 0.59 | 124 |
| Africa | Southeast Asia | Mali | Cambodia | 605 | 43,764 | 2.02 | 0 | 0.04 | 0.57 | 120 |
| Africa | Southeast Asia | Mali | Laos | 168 | 7,056 | 2.99 | 0 | 0.06 | 0.56 | 114 |
| Africa | Southeast Asia | Mali | Myanmar | 141 | 4,788 | 8.02 | 0 | 0.14 | 0.69 | 144 |
| Africa | Southeast Asia | Mali | Thailand | 224 | 11,760 | 6 | 0 | 0.11 | 0.58 | 120 |
| Africa | Southeast Asia | Mali | Vietnam | 180 | 8,064 | 2.24 | 0 | 0.04 | 0.53 | 108 |
| Africa | Southeast Asia | Senegal | Bangladesh | 176 | 5,895 | 3.26 | 0 | 0.03 | 0.55 | 115 |
| Africa | Southeast Asia | Senegal | Cambodia | 652 | 68,251 | 1.88 | 0 | 0.03 | 0.47 | 99 |
| Africa | Southeast Asia | Senegal | Laos | 215 | 11,004 | 1.31 | 0 | 0.03 | 0.65 | 138 |
| Africa | Southeast Asia | Senegal | Myanmar | 188 | 7,467 | 7.31 | 0 | 0.11 | 0.65 | 133 |
| Africa | Southeast Asia | Senegal | Thailand | 271 | 18,340 | 3.14 | 0 | 0.06 | 0.55 | 107 |
| Africa | Southeast Asia | Senegal | Vietnam | 227 | 12,576 | 1.41 | 0 | 0.03 | 0.69 | 145 |
| Africa | Southeast Asia | The Gambia | Bangladesh | 102 | 2,565 | 2.61 | 0 | 0.03 | 0.46 | 97 |
| Africa | Southeast Asia | The Gambia | Cambodia | 578 | 29,697 | 3.09 | 0 | 0.05 | 0.39 | 82 |
| Africa | Southeast Asia | The Gambia | Laos | 141 | 4,788 | 2.32 | 0 | 0.04 | 0.52 | 109 |
| Africa | Southeast Asia | The Gambia | Myanmar | 114 | 3,249 | 10.62 | 0 | 0.16 | 0.48 | 97 |
| Africa | Southeast Asia | The Gambia | Thailand | 197 | 7,980 | 5.56 | 0 | 0.09 | 0.47 | 99 |
| Africa | Southeast Asia | The Gambia | Vietnam | 153 | 5,472 | 3.86 | 0 | 0.06 | 0.48 | 102 |
| Africa | Oceania | DR of the Congo | PNG | 142 | 3,952 | 0.66 | 0 | 0.01 | 0.87 | 182 |
| Africa | Oceania | Ghana | PNG | 600 | 20,831 | 0.48 | 0 | 0.01 | 0.96 | 189 |
| Africa | Oceania | Guinea | PNG | 134 | 3,400 | 0.88 | 0 | 0.01 | 0.83 | 176 |
| Africa | Oceania | Malawi | PNG | 391 | 12,138 | 0.33 | 0 | 0 | 0.66 | 140 |
| Africa | Oceania | Mali | PNG | 122 | 3,192 | 0.5 | 0 | 0.01 | 3.57 | 749 |
| Africa | Oceania | Senegal | PNG | 169 | 4,978 | 0.26 | 0 | 0 | 1.38 | 288 |
| Africa | Oceania | The Gambia | PNG | 94 | 2,109 | 1.09 | 0 | 0.02 | 0.86 | 181 |
| Southeast Asia | Southeast Asia | Bangladesh | Cambodia | 566 | 23,445 | 4.08 | 0 | 0.04 | 0.46 | 94 |
| Southeast Asia | Southeast Asia | Bangladesh | Laos | 129 | 3,780 | 3.7 | 0 | 0.04 | 0.49 | 102 |
| Southeast Asia | Southeast Asia | Bangladesh | Myanmar | 102 | 2,565 | 7.99 | 0 | 0.07 | 0.52 | 105 |
| Southeast Asia | Southeast Asia | Bangladesh | Thailand | 185 | 6,300 | 6.19 | 0 | 0.05 | 0.51 | 106 |
| Southeast Asia | Southeast Asia | Bangladesh | Vietnam | 141 | 4,320 | 5 | 0 | 0.04 | 0.44 | 92 |
| Southeast Asia | Southeast Asia | Cambodia | Laos | 605 | 43,764 | 8.95 | 0 | 0.15 | 1.41 | 212 |
| Southeast Asia | Southeast Asia | Cambodia | Myanmar | 578 | 29,697 | 15.73 | 0 | 0.17 | 0.5 | 94 |
| Southeast Asia | Southeast Asia | Cambodia | Thailand | 661 | 72,940 | 23.08 | 0.2 | 1 | 3.34 | 294 |
| Southeast Asia | Southeast Asia | Cambodia | Vietnam | 617 | 50,016 | 19.22 | 0.06 | 0.39 | 1.4 | 188 |
| Southeast Asia | Southeast Asia | Laos | Myanmar | 141 | 4,788 | 10.07 | 0 | 0.11 | 0.45 | 89 |
| Southeast Asia | Southeast Asia | Laos | Thailand | 224 | 11,760 | 8.22 | 0 | 0.14 | 1.25 | 213 |
| Southeast Asia | Southeast Asia | Laos | Vietnam | 180 | 8,064 | 12.85 | 0 | 0.18 | 1.02 | 152 |
| Southeast Asia | Southeast Asia | Myanmar | Thailand | 197 | 7,980 | 41.37 | 0.01 | 0.55 | 0.94 | 134 |
| Southeast Asia | Southeast Asia | Myanmar | Vietnam | 153 | 5,472 | 17.05 | 0 | 0.16 | 0.54 | 104 |
| Southeast Asia | Southeast Asia | Thailand | Vietnam | 236 | 13,440 | 20.52 | 0 | 0.2 | 0.75 | 126 |
| Southeast Asia | Oceania | Bangladesh | PNG | 83 | 1,710 | 0.53 | 0 | 0.01 | 0.53 | 112 |
| Southeast Asia | Oceania | Cambodia | PNG | 558 | 19,277 | 0.48 | 0 | 0.01 | 0.44 | 92 |
| Southeast Asia | Oceania | Laos | PNG | 121 | 3,108 | 0.13 | 0 | 0 | 1.26 | 266 |
| Southeast Asia | Oceania | Myanmar | PNG | 94 | 2,109 | 0.14 | 0 | 0 | 0.63 | 133 |
| Southeast Asia | Oceania | Thailand | PNG | 177 | 5,180 | 0.29 | 0 | 0 | 0.69 | 147 |
| Southeast Asia | Oceania | Vietnam | PNG | 133 | 3,552 | 0.48 | 0 | 0 | 0.29 | 62 |
