# Supplementary material for: Identity-by-descent analyses for measuring population dynamics and selection in recombining pathogens
Source: PLoS Genet. 2018 May 23;14(5):e1007279. doi: 10.1371/journal.pgen.1007279 (PMC5988311; doi:10.1371/journal.pgen.1007279)
Supplement: S10 Table — The IBD proportion/XiR test statistic for the SNP with the largest–log10(p-value) within the selection interval is provided. (DOCX) [file pgen.1007279.s022.docx]

**S10 Table. The top 5 selection signals within each country for the Pf3k dataset.** The IBD proportion/X_iR_ test statistic for the SNP with the largest –log_10_(p-value) within the selection interval is provided.

| **Country** | **Chromosome** | **Start** | **End** | **IBD percentage** | **X_iR_** |
| --- | --- | --- | --- | --- | --- |
| DR of the Congo | 6 | 1040011 | 1292764 | 1.34 | 315.48 |
| DR of the Congo | 7 | 383527 | 688642 | 0.37 | 40.67 |
| DR of the Congo | 10 | 1421301 | 1570694 | 0.24 | 31.95 |
| DR of the Congo | 11 | 1950959 | 2003228 | 0.26 | 10.67 |
| DR of the Congo | 12 | 761260 | 909881 | 0.5 | 39.51 |
| Ghana | 2 | 756399 | 862030 | 0.27 | 39.44 |
| Ghana | 6 | 1070251 | 1294804 | 0.84 | 70.96 |
| Ghana | 7 | 210646 | 699826 | 0.13 | 77.21 |
| Ghana | 10 | 1350374 | 1571407 | 0.41 | 28.63 |
| Ghana | 12 | 721251 | 968375 | 0.53 | 69.44 |
| Guinea | 4 | 614998 | 698534 | 1.03 | 48.44 |
| Guinea | 6 | 1050336 | 1294804 | 1.39 | 269.69 |
| Guinea | 7 | 350609 | 659941 | 0.99 | 79.74 |
| Guinea | 12 | 760468 | 1029068 | 1.52 | 92.96 |
| Guinea | 12 | 1745700 | 1897010 | 0.67 | 30.13 |
| Malawi | 6 | 1081929 | 1294804 | 1 | 116.74 |
| Malawi | 8 | 372767 | 779813 | 0.69 | 150.8 |
| Malawi | 10 | 1400002 | 1570653 | 0.36 | 79.86 |
| Malawi | 11 | 1790115 | 2003228 | 0.45 | 18.07 |
| Malawi | 12 | 780748 | 1079903 | 0.71 | 68.74 |
| Mali | 6 | 1001323 | 1292769 | 3.82 | 79.33 |
| Mali | 7 | 360243 | 679897 | 3.24 | 109.95 |
| Mali | 10 | 1430096 | 1571407 | 1.03 | 46.47 |
| Senegal | 2 | 720032 | 861423 | 0.72 | 4.68 |
| Senegal | 3 | 901428 | 993530 | 0.72 | 5.45 |
| Senegal | 4 | 615405 | 748410 | 2.14 | 9.74 |
| Senegal | 6 | 1050336 | 1292769 | 6.62 | 323.28 |
| Senegal | 7 | 252368 | 619957 | 3.01 | 74.82 |
| The Gambia | 4 | 540033 | 779909 | 7.64 | 1447.64 |
| The Gambia | 4 | 930011 | 1143943 | 2.07 | 19.4 |
| The Gambia | 7 | 331248 | 719023 | 2.51 | 50.01 |
| The Gambia | 8 | 105218 | 219366 | 2.07 | 12.62 |
| The Gambia | 8 | 420318 | 649599 | 2.57 | 27.68 |
| Bangladesh | 4 | 620279 | 679065 | 0.71 | 19.87 |
| Bangladesh | 6 | 1191624 | 1294070 | 1.01 | 95.06 |
| Bangladesh | 7 | 175076 | 718355 | 1.62 | 69.07 |
| Bangladesh | 8 | 467370 | 580545 | 2.02 | 695.78 |
| Bangladesh | 12 | 766623 | 989679 | 0.91 | 99.4 |
| Cambodia | 8 | 420692 | 979511 | 8.94 | 64.92 |
| Cambodia | 10 | 550118 | 799419 | 6.8 | 38.57 |
| Cambodia | 13 | 1901748 | 2389910 | 9.26 | 73.76 |
| Cambodia | 14 | 35796 | 999557 | 4.92 | 46.27 |
| Cambodia | 14 | 1250576 | 1999655 | 6.8 | 65.82 |
| Laos | 1 | 93378 | 199493 | 2.81 | 25.87 |
| Laos | 3 | 71014 | 359186 | 3.18 | 68.25 |
| Laos | 4 | 540009 | 899970 | 3.82 | 201.13 |
| Laos | 8 | 425234 | 599944 | 3.56 | 55.12 |
| Laos | 13 | 75051 | 139950 | 3.18 | 71.22 |
| Myanmar | 4 | 545154 | 697350 | 7.52 | 76.87 |
| Myanmar | 6 | 1020329 | 1294426 | 8.15 | 110.64 |
| Myanmar | 7 | 450016 | 739678 | 4.82 | 36.25 |
| Myanmar | 11 | 1890328 | 2003228 | 4.26 | 50.42 |
| Myanmar | 12 | 631425 | 895415 | 10.34 | 94.4 |
| Thailand | 6 | 1002288 | 1294426 | 6.1 | 72.33 |
| Thailand | 7 | 405600 | 679897 | 7.87 | 80.05 |
| Thailand | 8 | 415327 | 698268 | 13.71 | 80.57 |
| Thailand | 11 | 1910597 | 2003233 | 6.55 | 81.83 |
| Thailand | 12 | 701638 | 977430 | 9.48 | 90.67 |
| Vietnam | 4 | 540009 | 679043 | 6.07 | 45.38 |
| Vietnam | 6 | 742857 | 959371 | 5.26 | 37.65 |
| Vietnam | 9 | 79451 | 498806 | 5.26 | 38.38 |
| Vietnam | 11 | 1900435 | 2003233 | 6.1 | 100.19 |
| Vietnam | 12 | 561246 | 1246071 | 6.71 | 113.73 |
| PNG | 5 | 803678 | 1099386 | 1.8 | 10.89 |
| PNG | 7 | 357431 | 849589 | 6.76 | 131.64 |
| PNG | 11 | 1900435 | 2001345 | 2.1 | 9.71 |
| PNG | 12 | 750192 | 999517 | 2.25 | 25.58 |
| PNG | 12 | 1680129 | 2162665 | 2.4 | 17.74 |
