# Supplementary material for: Identity-by-descent analyses for measuring population dynamics and selection in recombining pathogens
Source: PLoS Genet. 2018 May 23;14(5):e1007279. doi: 10.1371/journal.pgen.1007279 (PMC5988311; doi:10.1371/journal.pgen.1007279)
Supplement: S11 Table — (DOCX) [file pgen.1007279.s023.docx]

**S11 Table. List of 32 PlasmoDB genes within the selection interval chr6:1,102,005-1,283,312.**

| **Chromosome** | **Start** | **End** | **Strand** | **Gene ID** | **Gene Alias** |
| --- | --- | --- | --- | --- | --- |
| Pf3D7_06_v3 | 1101734 | 1102852 | - | PF3D7_0627500 | DJ1 |
| Pf3D7_06_v3 | 1104015 | 1105199 | + | PF3D7_0627600 | C6KTB2 |
| Pf3D7_06_v3 | 1108197 | 1111640 | + | PF3D7_0627700 | C6KTB3 |
| Pf3D7_06_v3 | 1114544 | 1117537 | - | PF3D7_0627800 | ACS |
| Pf3D7_06_v3 | 1119587 | 1120858 | + | PF3D7_0627900 | RPP29 |
| Pf3D7_06_v3 | 1121690 | 1122211 | + | PF3D7_0628000 | PTPS |
| Pf3D7_06_v3 | 1122801 | 1153664 | - | PF3D7_0628100 | C6KTB7 |
| Pf3D7_06_v3 | 1159862 | 1169080 | + | PF3D7_0628200 | PK4 |
| Pf3D7_06_v3 | 1170813 | 1173696 | - | PF3D7_0628300 | CEPT |
| Pf3D7_06_v3 | 1176265 | 1178587 | + | PF3D7_0628400 | C0H4H7 |
| Pf3D7_06_v3 | 1179053 | 1180003 | - | PF3D7_0628500 | C6KTC0 |
| Pf3D7_06_v3 | 1180573 | 1182353 | - | PF3D7_0628600 | C6KTC1 |
| Pf3D7_06_v3 | 1183896 | 1185617 | + | PF3D7_0628700 | C6KTC2 |
| Pf3D7_06_v3 | 1185996 | 1188644 | - | PF3D7_0628800 | GATB |
| Pf3D7_06_v3 | 1189472 | 1193978 | + | PF3D7_0628900 | C6KTC4 |
| Pf3D7_06_v3 | 1194304 | 1195179 | - | PF3D7_0629000 | C6KTC5 |
| Pf3D7_06_v3 | 1196041 | 1198038 | - | PF3D7_0629100 | NAPRT |
| Pf3D7_06_v3 | 1199853 | 1200995 | - | PF3D7_0629200 | C6KTC7 |
| Pf3D7_06_v3 | 1205190 | 1207781 | + | PF3D7_0629300 | UIS10 |
| Pf3D7_06_v3 | 1210420 | 1212762 | + | PF3D7_0629400 | C6KTC9 |
| Pf3D7_06_v3 | 1213948 | 1216005 | - | PF3D7_0629500 | AAT1 |
| Pf3D7_06_v3 | 1218307 | 1219251 | + | PF3D7_0629600 | C6KTD1 |
| Pf3D7_06_v3 | 1221941 | 1242922 | + | PF3D7_0629700 | SET1 |
| Pf3D7_06_v3 | 1243580 | 1247219 | - | PF3D7_0629800 | C6KTD3 |
| Pf3D7_06_v3 | 1250412 | 1251651 | + | PF3D7_0629900 | C6KTD4 |
| Pf3D7_06_v3 | 1252052 | 1253985 | - | PF3D7_0630000 | C6KTD5 |
| Pf3D7_06_v3 | 1254907 | 1256940 | - | PF3D7_0630100 | C6KTD6 |
| Pf3D7_06_v3 | 1258880 | 1259939 | + | PF3D7_0630200 | PSOP6 |
| Pf3D7_06_v3 | 1260398 | 1269596 | - | PF3D7_0630300 | C6KTD8 |
| Pf3D7_06_v3 | 1272363 | 1273532 | - | PF3D7_0630400 | C6KTD9 |
| Pf3D7_06_v3 | 1276329 | 1277825 | + | PF3D7_0630500 | YTM1 |
| Pf3D7_06_v3 | 1279820 | 1283246 | + | PF3D7_0630600 | C6KTE1 |
