# Supplementary material for: Identity-by-descent analyses for measuring population dynamics and selection in recombining pathogens
Source: PLoS Genet. 2018 May 23;14(5):e1007279. doi: 10.1371/journal.pgen.1007279 (PMC5988311; doi:10.1371/journal.pgen.1007279)
Supplement: S12 Table — (DOCX) [file pgen.1007279.s024.docx]

**S12 Table. List of 94 PlasmoDB genes within the selection interval chr12:700,000-1,100,000.**

| **Chromosome** | **Start** | **End** | **Strand** | **Gene ID** | **Gene Alias** |
| --- | --- | --- | --- | --- | --- |
| Pf3D7_12_v3 | 700531 | 702211 | + | PF3D7_1217900 | PFL0865W |
| Pf3D7_12_v3 | 703895 | 704953 | + | PF3D7_1218000 | TRAMP |
| Pf3D7_12_v3 | 706227 | 708196 | + | PF3D7_1218100 | PFL0875W |
| Pf3D7_12_v3 | 708936 | 715665 | - | PF3D7_1218200 | PFL0880C |
| Pf3D7_12_v3 | 717955 | 719820 | + | PF3D7_1218300 | AP2-MU |
| Pf3D7_12_v3 | 720886 | 723448 | - | PF3D7_1218400 | PFL0890C |
| Pf3D7_12_v3 | 725841 | 729837 | - | PF3D7_1218500 | DYN3 |
| Pf3D7_12_v3 | 732174 | 734959 | - | PF3D7_1218600 | RRS |
| Pf3D7_12_v3 | 736335 | 737825 | - | PF3D7_1218700 | PFL0905C |
| Pf3D7_12_v3 | 738357 | 740255 | - | PF3D7_1218800 | PSOP17 |
| Pf3D7_12_v3 | 740994 | 743147 | - | PF3D7_1218900 | PFL0920C |
| Pf3D7_12_v3 | 746002 | 754926 | + | PF3D7_1219000 | FRM2 |
| Pf3D7_12_v3 | 757649 | 763642 | + | PF3D7_1219100 | PFL0930W |
| Pf3D7_12_v3 | 764448 | 765151 | + | PF3D7_1219200 | RIF |
| Pf3D7_12_v3 | 766654 | 774197 | - | PF3D7_1219300 | VAR |
| Pf3D7_12_v3 | 776510 | 779933 | - | PF3D7_1219400 | VAR |
| Pf3D7_12_v3 | 784018 | 784830 | + | PF3D7_1219500 | VAR |
| Pf3D7_12_v3 | 785571 | 790673 | - | PF3D7_1219600 | ATPase2 |
| Pf3D7_12_v3 | 792877 | 793449 | - | PF3D7_1219700 | RKIP |
| Pf3D7_12_v3 | 795157 | 795318 | + | PF3D7_1219800 | C6S3K7 |
| Pf3D7_12_v3 | 796500 | 797183 | + | PF3D7_1219900 | PFL0960W |
| Pf3D7_12_v3 | 798304 | 799611 | - | PF3D7_1220000 | PFL0965C |
| Pf3D7_12_v3 | 802544 | 804400 | + | PF3D7_1220100 | PRP17 |
| Pf3D7_12_v3 | 804963 | 806698 | - | PF3D7_1220200 | PFL0973C |
| Pf3D7_12_v3 | 807560 | 816204 | + | PF3D7_1220300 | PFL0975W |
| Pf3D7_12_v3 | 816909 | 818468 | + | PF3D7_1220400 | DRN1 |
| Pf3D7_12_v3 | 818703 | 819659 | - | PF3D7_1220500 | TSR3 |
| Pf3D7_12_v3 | 820586 | 822198 | + | PF3D7_1220600 | PFL0990W |
| Pf3D7_12_v3 | 822958 | 824204 | - | PF3D7_1220700 | PFL0995C |
| Pf3D7_12_v3 | 825697 | 827712 | - | PF3D7_1220800 | PFL1000C |
| Pf3D7_12_v3 | 831252 | 832052 | - | PF3D7_1220900 | HP1 |
| Pf3D7_12_v3 | 836912 | 843901 | - | PF3D7_1221000 | SET10 |
| Pf3D7_12_v3 | 847437 | 849183 | + | PF3D7_1221100 | PFL1015W |
| Pf3D7_12_v3 | 849840 | 850652 | + | PF3D7_1221200 | PFL1020W |
| Pf3D7_12_v3 | 851012 | 854739 | - | PF3D7_1221300 | PFL1025C |
| Pf3D7_12_v3 | 857097 | 858671 | + | PF3D7_1221400 | ALV3 |
| Pf3D7_12_v3 | 860262 | 862790 | + | PF3D7_1221500 | PFL1035W |
| Pf3D7_12_v3 | 863810 | 865186 | + | PF3D7_1221600 | PFL1040W |
| Pf3D7_12_v3 | 865726 | 868581 | + | PF3D7_1221700 | PFL1045W |
| Pf3D7_12_v3 | 869260 | 871368 | + | PF3D7_1221800 | PFL1050W |
| Pf3D7_12_v3 | 872361 | 874940 | - | PF3D7_1221900 | PFL1055C |
| Pf3D7_12_v3 | 876916 | 878625 | - | PF3D7_1222000 | PFL1060C |
| Pf3D7_12_v3 | 879630 | 881186 | - | PF3D7_1222100 | PFL1065C |
| Pf3D7_12_v3 | 881970 | 882114 | - | PF3D7_1222200 | snoR15 |
| Pf3D7_12_v3 | 883567 | 886032 | - | PF3D7_1222300 | HSP90 |
| Pf3D7_12_v3 | 890581 | 898257 | + | PF3D7_1222400 | ApiAP2 |
| Pf3D7_12_v3 | 899198 | 901076 | - | PF3D7_1222500 | DPH6 |
| Pf3D7_12_v3 | 907203 | 914501 | + | PF3D7_1222600 | AP2-G |
| Pf3D7_12_v3 | 918722 | 919336 | + | PF3D7_1222700 | GAP45 |
| Pf3D7_12_v3 | 920790 | 922416 | - | PF3D7_1222800 | PFL1095C |
| Pf3D7_12_v3 | 923527 | 925198 | + | PF3D7_1222900 | PFL1100W |
| Pf3D7_12_v3 | 925375 | 926249 | - | PF3D7_1223000 | PFL1105C |
| Pf3D7_12_v3 | 927825 | 929991 | - | PF3D7_1223100 | PKAr |
| Pf3D7_12_v3 | 932917 | 934281 | + | PF3D7_1223200 | PFL1115W |
| Pf3D7_12_v3 | 934971 | 938639 | - | PF3D7_1223300 | GyrA |
| Pf3D7_12_v3 | 941125 | 946745 | + | PF3D7_1223400 | PFL1125W |
| Pf3D7_12_v3 | 946864 | 960969 | - | PF3D7_1223500 | PFL1130C |
| Pf3D7_12_v3 | 962631 | 967491 | - | PF3D7_1223600 | PFL1135C |
| Pf3D7_12_v3 | 968980 | 970073 | + | PF3D7_1223700 | VIT |
| Pf3D7_12_v3 | 971268 | 972194 | + | PF3D7_1223800 | YHM2 |
| Pf3D7_12_v3 | 972710 | 973393 | - | PF3D7_1223900 | PFF0245W |
| Pf3D7_12_v3 | 974372 | 975541 | + | PF3D7_1224000 | GCH1 |
| Pf3D7_12_v3 | 976820 | 977889 | - | PF3D7_1224100 | PFL1160C |
| Pf3D7_12_v3 | 981301 | 984475 | + | PF3D7_1224200 | PFL1165W |
| Pf3D7_12_v3 | 988628 | 991255 | + | PF3D7_1224300 | PABP |
| Pf3D7_12_v3 | 994255 | 996438 | + | PF3D7_1224400 | PFL1175W |
| Pf3D7_12_v3 | 998353 | 999275 | + | PF3D7_1224500 | ASF1 |
| Pf3D7_12_v3 | 1000679 | 1001945 | - | PF3D7_1224600 | CCHL |
| Pf3D7_12_v3 | 1003201 | 1007586 | - | PF3D7_1224700 | PFL1190C |
| Pf3D7_12_v3 | 1009289 | 1010251 | + | PF3D7_1224800 | PFL1195W |
| Pf3D7_12_v3 | 1010832 | 1011342 | - | PF3D7_1224900 | SF3B6 |
| Pf3D7_12_v3 | 1012310 | 1017848 | - | PF3D7_1225000 | PFL1205C |
| Pf3D7_12_v3 | 1018506 | 1024008 | + | PF3D7_1225100 | PFL1210W |
| Pf3D7_12_v3 | 1024455 | 1028018 | - | PF3D7_1225200 | PFL1215C |
| Pf3D7_12_v3 | 1029862 | 1031388 | + | PF3D7_1225300 | PFL1220W |
| Pf3D7_12_v3 | 1031825 | 1032565 | - | PF3D7_1225400 | PFL1225C |
| Pf3D7_12_v3 | 1033551 | 1035112 | + | PF3D7_1225500 | PFL1230W |
| Pf3D7_12_v3 | 1035261 | 1038155 | - | PF3D7_1225600 | PFL1235C |
| Pf3D7_12_v3 | 1039410 | 1043915 | - | PF3D7_1225700 | PFL1240C |
| Pf3D7_12_v3 | 1045995 | 1049831 | + | PF3D7_1225800 | UBA1 |
| Pf3D7_12_v3 | 1050280 | 1052400 | - | PF3D7_1225900 | PFL1250C |
| Pf3D7_12_v3 | 1053296 | 1056282 | - | PF3D7_1226000 | PFL1255C |
| Pf3D7_12_v3 | 1058202 | 1059152 | + | PF3D7_1226100 | HAD3 |
| Pf3D7_12_v3 | 1059718 | 1061469 | - | PF3D7_1226200 | PFL1265C |
| Pf3D7_12_v3 | 1065796 | 1066683 | + | PF3D7_1226300 | HAD2 |
| Pf3D7_12_v3 | 1067470 | 1071309 | - | PF3D7_1226400 | PFL1275C |
| Pf3D7_12_v3 | 1072459 | 1076976 | + | PF3D7_1226500 | PFL1280W |
| Pf3D7_12_v3 | 1077868 | 1078776 | - | PF3D7_1226600 | PCNA2 |
| Pf3D7_12_v3 | 1081198 | 1083108 | + | PF3D7_1226700 | RRP9 |
| Pf3D7_12_v3 | 1085387 | 1087264 | + | PF3D7_1226800 | ATX3 |
| Pf3D7_12_v3 | 1088536 | 1090023 | - | PF3D7_1226900 | PV2 |
| Pf3D7_12_v3 | 1092160 | 1092651 | - | PF3D7_1227000 | PFL1305C |
| Pf3D7_12_v3 | 1093543 | 1095771 | - | PF3D7_1227100 | DH60 |
| Pf3D7_12_v3 | 1098129 | 1104223 | + | PF3D7_1227200 | KCh1 |
