# Supplementary material for: Identity-by-descent analyses for measuring population dynamics and selection in recombining pathogens
Source: PLoS Genet. 2018 May 23;14(5):e1007279. doi: 10.1371/journal.pgen.1007279 (PMC5988311; doi:10.1371/journal.pgen.1007279)
Supplement: S1 Methods — (DOCX) [file pgen.1007279.s025.docx]

**S1 Methods**

**Identity-by-descent analyses for measuring population dynamics and selection in recombining pathogens**

Lyndal Henden^1,2^, Stuart Lee^3,4^, Ivo Mueller^1,2^, Alyssa Barry^1,2^, Melanie Bahlo^1,2,^*

1. Population Health and Immunity Division, The Walter and Eliza Hall Institute of Medical Research, Parkville VIC, Australia
2. Department of Medical Biology, University of Melbourne, Parkville VIC, Australia
3. Department of Econometrics and Business Statistics, Monash University, Clayton VIC, Australia
4. Molecular Medicine Division, The Walter and Eliza Hall Institute of Medical Research, Parkville VIC, Australia

* Corresponding author

E-mail: bahlo@wehi.edu.au (MB)

**Algorithm 1 – simulating a haplotype**

let *N* equal the total number of SNPs;

let *p_i_* denote the population allele frequency at SNP *i*;

for *i* in 1 to *N* {

generate *y* ~ Unif(0,1);

if *y < p_i_* {

allele A is chosen for SNP *i;*

} else {

allele B is chosen for SNP *i;*

}

}

**Algorithm 2 – simulating recombination**

Let *L* denote the length of chromosome 12 in Morgans;

Let *X_1_* and *X_2_* denote two homologous chromosomes;

Generate *y* ~ Unif(0,1);

If *y < 0.5* {

*X_1_* is chosen as the start chromosome;

} else {

*X_2_* is chosen as the start chromosome;

}

generate *z ~* Exp(1);

let *t = z;*

if *t < L* {

recombination occurs at *t;*

} else {

recombination does not occur and the offspring inherits a non-recombined chromosome;

}

while *t < L* {

generate *z ~* Exp(1);

*t = t + z;*

if *t < L* {

recombination at *t;*

} else {

stop, not more recombination;

}

}

**Processing of Papua New Guinea Dataset**

Picard Tools version 2.2.1 was used with the MarkIlluminaAdapters module to soft clip reads containing adapter sequences (Picard Tools, 2016). Following this paired-end reads were mapped to the Pf3D7 v3 reference genome with bwa-mem with the mark secondary hits option enabled (Li, 2013). The Genome Analysis Toolkit (GATK) version 3.5 was used with the RealignIndels walker to perform local realignment around intervals (DePristo et al., 2011) and Picard-tools MarkDuplicates module was used to remove PCR duplicates. Next, GATK’s BaseRecalibrator walker was used to correct base-quality scores using the entire *P. falciparum* genetic crosses version 1.0 data as known sites of variation (Miles et al., 2016). Quality assessment of the aligned BAM files were performed with FastQC version 0.10.1 (Andrews, 2010) and Picard Tools CollectAlignmentMetrics and CollectInsertSizeMetrics module. Finally, coverage analysis was performed using GATK’s Depth of Coverage walker with the conditions that reads had to have a mapping quality score of at least 20 and bases had to have a minimum quality of 20. As a result, 29 isolates were removed as less than 90% of their bases were not covered to at least 5 reads or did not map at all to the reference genome.

Variants were called using GATK’s HaplotypeCaller walker in gVCF mode and genotypes were jointly called using GATK’s GenotypeGVCF walker. Following this, variant quality score recalibration was performed for SNPs using the VariantRecalibrator walker. The *P. falciparum* genetic crosses data was used as training data and the calibration model was trained using the QD, MQ, FS, SOR and DP tags in the VCF file. Annotation was performed using snpEff version 4.1 and a custom annotation was added to the final VCF file using the RegionType annotations obtained from the *P. falciparum* genetic crosses data using bcftools version 1.1 (Cingolani et al., 2012; Li et al., 2009). We applied the same filtering procedure to the isolates and SNP calls as for the MalariaGEN pf3k field isolates. The final VCF file consisted of 38 isolates and 29,631 SNPs.

**Hidden Markov Model for IBD Detection**

A hidden Markov model (HMM) is a probabilistic model that determines the most likely sequence of events that gave rise to an observation set. In order to do so it requires an observation set, a state space and a number of probability distributions. Here we describe our first order, continuous time, HMM that we use to infer IBD between pairs of isolates, with or without multiple infections. This model is based on the model described by Henden et al. (2016).

**Observations**

Our model uses unphased genotype data from biallelic SNPs as its observation set, where genotypes are referred to as

| Haploid genotype | Diploid genotype | Genotype status |
| --- | --- | --- |
| A | AA | Homozygous reference |
| a | aa | Homozygous alternative |
| - | Aa | Heterozygous |

**State space**

A key assumption of our model is that the input species is haploid. Therefore, an isolate with a single infection (MOI = 1) can only ever share either zero or one allele IBD with any other isolate, regardless of the second isolate’s infection status. In contrast, an isolate with multiple infections (MOI > 1) can share zero, one or at most two alleles IBD by descent with any other isolate. Allele sharing is restricted to two alleles IBD, when both isolates have MOI > 1, since we only consider biallelic SNPs.

**Initial probabilities**

The probabilities of being in each state are termed the initial probabilities and are denoted by ω_0_, ω_1_ and ω_2_. These probabilities are calculated by the method-of-moments approach described in Purcell et al. (2007). We note that ω_2_ = 0 when at least one isolate has MOI = 1.

**Transition probabilities**

The probabilities of transitioning from one state to another between adjacent SNPs are modeled as the transition probabilities. Our transition probabilities are functions of the state space, the initial probabilities, the genetic map distance *t* between adjacent SNPs (Morgans), the recombination rate *θ* and the number of meiosis *m* separating the pair of isolates. We assume recombination events follow a Poison distribution with rate *α*, therefore the time between recombination events follows an exponential distribution and as such we model the transition probabilities as

$$A= \left[ \begin{matrix} \omega_{0}+\omega_{1}e^{-\alpha t} & \omega_{1}(1-e^{-\alpha t}) \\ \omega_{0}(1-e^{-\alpha t}) & \omega_{1}+\omega_{0}e^{-\alpha t} \end{matrix} \right]$$

when at least one isolate has MOI = 1, and

$$A=\left[ \begin{matrix} 1-\left( 1-e^{-\alpha t} \right)\omega_{1}-T_{0,2} & \left( 1-e^{-\alpha t} \right)\omega_{1} & T_{0,2} \\ \left( 1-e^{-\alpha t} \right)\omega_{0} & \left( 1-e^{-\alpha t} \right)\omega_{1}+e^{-\alpha t} & \left( 1-e^{-\alpha t} \right)\omega_{2} \\ T_{2,0} & \left( 1-e^{-\alpha t} \right)\omega_{1} & 1-\left( 1-e^{-\alpha t} \right)\omega_{1}+T_{2,0} \end{matrix} \right]$$

when both isolates have MOI > 1, where

$$T_{0,2}=\frac{e^{-\alpha\omega_{1}t}\omega_{2}}{\omega_{1}-1}+e^{-\alpha t}\omega_{1}+\frac{e^{-\alpha t}\omega_{0}\omega_{1}}{\omega_{1}-1}+\omega_{2}$$

and

$$T_{0,2}=\frac{e^{-\alpha\omega_{1}t}\omega_{0}}{\omega_{1}-1}+e^{-\alpha t}\omega_{1}+\frac{e^{-\alpha t}\omega_{2}\omega_{1}}{\omega_{1}-1}+\omega_{0}$$

The parameters *α* = -*m*ln(1 - *θ*) controls the frequency of transitions between states, where *m* is estimated as in Purcell et al. (2007) and *θ* is estimated by Ott (1999).

**Emission probabilities**

The emission probabilities model the probabilities of the observations from the state space and are functions of the observed genotypes, state space and population allele frequencies. Let *p_A_* denote the frequency of the reference allele and *p_a_* = 1 – *p_A_* denote the frequency of the alternative allele. Additionally let $G_{i}^{l}$ and $G_{i}^{k}$ denote the genotypes for individuals *l* and *k* at SNP *i*, and $G_{i}^{l,k}=\{G_{i}^{l},G_{i}^{k}\}$ denote the genotype pair. Also let *Zi* denote the number of alleles IBD at SNP *i*. By symmetry we get $\Pr(G_{i}^{l,k}|Z_{i}=z)=\Pr(G_{i}^{k,l}|Z_{i}=z)$ and the emission probabilities are given by

| MOI*^l^* | MOI*^k^* | $G_{i}^{l}$ | $G_{i}^{k}$ | $\Pr(G_{i}^{l,k}\vert Z_{i}=0)$ | $\Pr(G_{i}^{l,k}\vert Z_{i}=1)$ | $\Pr(G_{i}^{l,k}\vert Z_{i}=2)$ |
| --- | --- | --- | --- | --- | --- | --- |
| 1 | 1 | A | A | $p_{A}^{2}$ | $p_{A}$ | 0 |
| 1 | 1 | A | a | ${2p}_{A}p_{a}$ | 0 | 0 |
| >1 | 1 | AA | A | $p_{A}^{3}$ | $p_{A}^{2}$ | 0 |
| >1 | 1 | AA | A | $p_{A}^{2}p_{a}$ | 0 | 0 |
| >1 | 1 | Aa | A | ${2p}_{A}^{2}p_{a}$ | $p_{A}p_{a}$ | 0 |
| >1 | >1 | AA | AA | $p_{A}^{4}$ | $p_{A}^{3}$ | $p_{A}^{2}$ |
| >1 | >1 | AA | aa | ${2p}_{A}^{2}p_{a}^{2}$ | 0 | 0 |
| >1 | >1 | AA | Aa | ${4p}_{A}^{3}p_{a}$ | ${2p}_{A}^{2}p_{a}$ | 0 |
| >1 | >1 | Aa | aa | ${4p}_{A}^{2}p_{a}^{2}$ | $p_{A}^{2}p_{a}+p_{A}p_{a}^{2}$ | ${2p}_{A}p_{a}$ |

**Genotyping errors**

Let *ε* denote the genotyping error rate. Our model accounts for genotyping errors as in Albrechtsen et al. (2009) where the probability of the observed genotype given the true genotype for a MOI = 1 isolate is

| $\Pr(G_{i}^{l}\vert G_{i}^{l'})$ | $G_{i}^{l}=A$ | $G_{i}^{l}=a$ |
| --- | --- | --- |
| $G_{i}^{l'}=A$ | 1 – *ε* | *ε* |
| $G_{i}^{l'}=a$ | *ε* | 1 – *ε* |

and for a MOI > 1 isolate

| $\Pr(G_{i}^{l}\vert G_{i}^{l'})$ | $G_{i}^{l}=AA$ | $G_{i}^{l}=Aa$ | $G_{i}^{l}=aa$ |
| --- | --- | --- | --- |
| $G_{i}^{l'}=AA$ | (1 – *ε*)^2^ | 2(1 – *ε*) *ε* | *ε^2^* |
| $G_{i}^{l'}=Aa$ | (1 – *ε*) *ε* | (1 – *ε*) ^2^ + *ε^2^* | (1 – *ε*) *ε* |
| $G_{i}^{l'}=aa$ | *ε^2^* | 2(1 – *ε*) *ε* | (1 – *ε*)^2^ |

**Missing data**

We account for missing genotypes at a SNP by summing the emission probabilities over all possible combinations of genotype pairs.

**References**

Polley, S. D. & Conway, D. J. (2001) Strong diversifying selection on domains of the Plasmodium falciparum apical membrane antigen 1 gene. *Genetics* **158,** 1505–1512

Mu, J. *et al.* (2010) Plasmodium falciparum genome-wide scans for positive selection, recombination hot spots and resistance to antimalarial drugs. *Nat. Genet.* **42,** 268–271

Weedall, G. D., Preston, B. M. J., Thomas, A. W., Sutherland, C. J. & Conway, D. J. (2007) Differential evidence of natural selection on two leading sporozoite stage malaria vaccine candidate antigens. *Int. J. Parasitol.* **37,** 77–85

Nwakanma, D. C. *et al.* (2014) Changes in malaria parasite drug resistance in an endemic population over a 25-year period with resulting genomic evidence of selection. *J. Infect. Dis.* **209,** 1126–1135

Baum, J., Thomas, A. W. & Conway, D. J. (2003) Evidence for diversifying selection on erythrocyte-binding antigens of Plasmodium falciparum and P. vivax. *Genetics* **163,** 1327–1336

Conway, D. J. (1997) Natural selection on polymorphic malaria antigens and the search for a vaccine. *Parasitol. Today* **13,** 26–29

Miotto, O. *et al.* (2013) Multiple populations of artemisinin-resistant Plasmodium falciparum in Cambodia. *Nat. Genet.* **45,** 648–655

Tetteh, K. K. A. *et al.* (2009) Prospective identification of malaria parasite genes under balancing selection. *PLoS One* **4,** e5568

Picard Tools, viewed 27 July 2016, http://broadinstitute.github.io/picard/

Li, H. (2013) Aligning sequence reads, clone sequences and assembly contigs with BWA-MEM. arXiv:1303.3997

DePristo *et al.* (2011) A framework for variation discovery and genotyping using next-generation DNA sequencing data. *Nat Genet*, **43(5)**:491-498

Miles, A. *et al.* (2016) Indels, structural variation, and recombination drive genomic diversity in Plasmodium falciparum. *Genome Res*, **26**:1-12

Andrews, S. (2010) FastQC: A quality control tool for high throughput sequence data. Available online at http://www.bioinformatics.babraham.ac.uk/projects/fastqc

Cingolani, P. *et al.* (2012) A program for annotating and predicting the effects of single nucleotide polymorphisms, SnpEff: SNPs in the genome of Drosophila melanogaster strain w1118; iso-2; iso-3. *Fly*, **6(2)**:80-92

Li, H. *et al.* (2009) The sequence alignment/map format and SAMtools. *Bioinformatics*, **25(16)**:2078-2079

Albrechtsen, A . *et al.* (2009) Relatedness mapping and tracts of relatedness for genome-wide data in the presence of linkage disequilibrium*. Genet Epidemiol*, **33(3)**:266-274

Henden, L. Wakeham, D. Bahlo, M. (2016) XIBD: software for inferring pairwise identity by descent on the X chromosome. *Bioinformatics*, **32(15)**:2389-2391

Ott, J. (1999) *Introduction and basic genetic principles*. Johns Hopkins University Press, Baltimore, London

Purcell, S. *et al.* (2007) PLINK: a tool set for whole-genome association and population-based linkage analysis. *Am J Hum Genet*, **81(3)**:559-575
